# A Network Calibration Approach Improves the Accuracy and Long-Term Stability of a Low-Cost Air Quality Mesonet in New York City

**DOI:** 10.1021/acsestair.5c00205

**Published:** 2025-12-25

**Authors:** Ellie H. Hojeily, Jason M. Covert, Margaret J. Schwab, Clover Moore, Cheng-Hsuan Lu, Md. Aynul Bari, Scott D. Miller

**Affiliations:** † Atmospheric Sciences Research Center, 1084 University at Albany, Albany, New York 12226, United States; ‡ Joint Center for Satellite Data Assimilation, University Corporation for Atmospheric Research, Boulder, Colorado 80301, United States; § Department of Environmental and Sustainable Engineering, University at Albany, Albany, New York 12226, United States

**Keywords:** air quality, low-cost sensors, calibration, mesonet

## Abstract

A new calibration approach, the Network Calibration Algorithm (NCA), was developed and applied to low-cost sensors measuring PM_2.5_, O_3_, NO_2_, NO, and CO at 38 New York State Mesonet sites in the New York City Metropolitan Area. A single low-cost sensor package (the “keystone” package) was colocated alongside regulatory-grade (reference) instruments at the New York State Department of Environmental Conservation Queens College monitoring site for 16 months. For each pollutant, hourly data from the keystone package and reference instruments were used to train a single calibration model that was subsequently applied to all packages at field sites across the network. The calibration models included multiple linear regression (MLR) for CO and a hybrid approach that combined MLR with a Random Forest model for PM_2.5_, O_3_, NO_2_, and NO. The performance of the NCA-calibrated low-cost sensors was quantified using multiple evaluation data sets, with a focus on accuracy and long-term stability over the 16-month period. The performance statistics were consistent with or better than previous reports for similar low-cost sensors, and the NCA was able to compensate for sensor degradation and drift. Empirical estimates of the field limit of detection for each of the low-cost sensors are presented.

## Graphical abstract

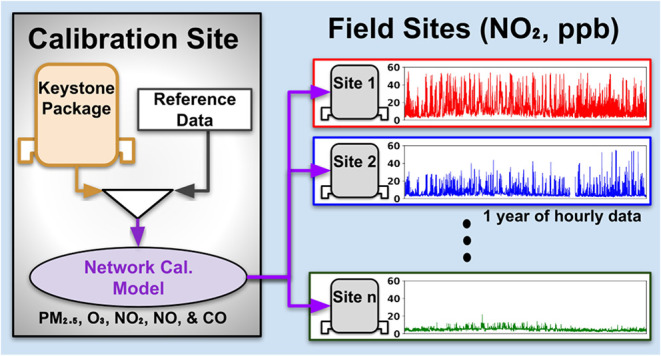

## 1 Introduction

Over the past decade, low-cost air quality sensors have emerged as a complement to sparsely located regulatory pollutant monitors, enabling dense deployments and finer-scale monitoring.^1–4^ Careful calibration of low-cost sensors is critical due to their sensitivities to ambient conditions (e.g., temperature and humidity) and other pollutants.^4–6^ Field calibrations, where low-cost sensors are temporarily colocated with reference-grade instruments, have been shown to produce accurate short-term calibrations;^7–9^ however, longer-term deployments (seasons to years) are susceptible to decreased accuracy due to seasonal meteorological and pollutant concentration variations and sensor degradation and drift.^5,10,11^ Periodic recalibrations could potentially mitigate these issues; however, the logistics and costs of this approach may be considerable, particularly for large networks, thereby undermining a common rationale for deploying low-cost sensors.^12–14^ Improved approaches to calibrate low-cost sensors that reduce costs while producing accurate and stable calibrations have potential to greatly increase their utility.^15^

The typical calibration approach for low-cost air quality sensors involves “individual” models (e.g., multiple linear regression or machine learning) for each pollutant sensor within each sensor package, determined from short-term (at least 30 days) colocation periods at a reference site, as recommended by the U.S. Environmental Protection Agency.^16,17^ In contrast, “general” or “network” approaches have been developed that aim to reduce or eliminate the need for colocations at a reference site. For example, a universal calibration model developed for the low-cost particulate matter (PM_2.5_) sensor used by PurpleAir can be applied across the United States without unique calibrations for each sensor.^18^ For trace gas pollutant sensors, Malings et al. (2019) developed a general calibration approach whereby models trained using the median output from a colocated subset of sensors were applied to all sensors across the network.^10^ Alternatively, Winter et al. (2025) eliminated the need for colocations altogether by using a network of nearby (but not colocated) reference site concentrations during periods when their reported pollutant concentrations were relatively homogeneous across the network.^12^ Generally, network approaches have shown better results than the typical calibration approach as they can leverage longer training data sets to build more representative models.^10,12^

The University at Albany (UAlbany) designed and built 60 low-cost sensor packages to measure PM_2.5_, ozone (O_3_), nitrogen dioxide (NO_2_), nitric oxide (NO), and carbon monoxide (CO), and installed them at 38 New York State Mesonet (NYSM)^19^ sites in the New York City Metropolitan Area between April 8, 2023 and August 31, 2024. The network design and integration with the NYSM are detailed in a companion paper (Miller et al. 2025).^20^ Here, we present a new network-based calibration, the Network Calibration Algorithm (NCA), whereby a single low-cost sensor package (the “keystone package”) was permanently colocated with reference monitors at the New York State Department of Environmental Conservation (NYSDEC) Queens College monitoring site. Fifteen months of hourly data from the keystone package were used as training data to develop a single calibration model per pollutant that was applied across the 38-site low-cost sensor network. The extensive training data set enabled the calibration models to account for seasonal variation of meteorological conditions and pollutant concentrations, and to compensate for degradation and drift of the low-cost sensors. Here, the NCA is described, including pollutant-specific calibration models, and its performance is quantified by comparison of NCA-calibrated low-cost sensors with reference monitors, with a focus on accuracy and long-term stability. In addition, empirical estimates of the field limit of detection (fLOD) for the calibrated sensors are presented.

## 2 Materials and Methods

### 2.1 UAlbany Low-Cost Sensor Package

The sensor package was designed and manufactured at UAlbany. Photos of the sensor package and a list of the low-cost sensors and reference instruments at the NYSDEC Queens College site are provided in Figure S1 and Table S1, respectively. PM_2.5_ was measured using a Plantower PMS5003 optical sensor (Plantower, Inc., China), and NO_2_, O_3_+NO_2_, NO, and CO were measured using the Alphasense B4 series electrochemical sensors (Alphasense, Inc., Essex, UK). Temperature and relative humidity (RH) were measured both inside the sensor manifold and outside the package (Figure S1). Each package was assigned a serial number starting with “AQA20” and ending with a sequentially increasing two-digit number. Sixty sensor packages were fabricated between November 2022 and March 2024. Raw data, including meteorological and pollutant variables, were collected at 5-s sample period. Further details on the package design and integration into the NYSM are provided in Miller et al. (2025).^20^

### 2.2 NYSDEC Queens College Calibration Site

The NCA was developed using data collected at the NYSDEC Queens College monitoring site, located in Queens, NY (Figure 1a, yellow star). The site consisted of a trailer located adjacent to athletic fields and a parking lot, with Interstate Highway 495 located approximately 400 m to the north (Figure S2). The site is part of the National Core Network, measuring PM_2.5_, O_3_, NO_2_, NO, and CO.^21^ Additional information about the site and instrumentation is provided in Table S1. Reference data were retrieved from the U.S. Environmental Protection Agency (EPA) Air Quality System (www.epa.gov/aqs).

**Figure 1:**
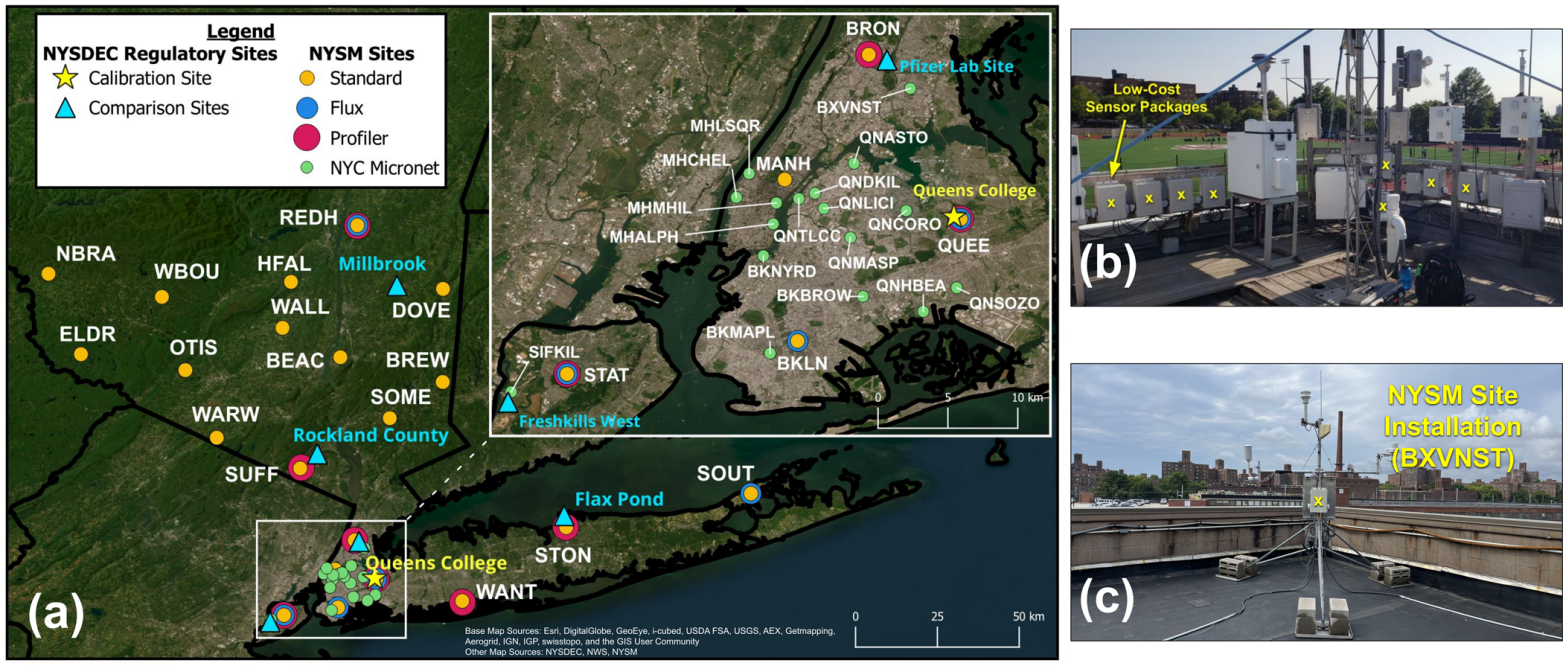
(a) Site locations where air quality sensor packages were deployed within the NYC Metropolitan Area (NYCMA). New York State Mesonet sites are indicated according to site type: standard meteorological station (4-letter acronym, yellow); urban micronet (6-letter acronym, green); eddy covariance flux site (blue); and profiler site (red). Inset shows sites within the 5 NYC boroughs in greater detail. The NYSDEC Queens College calibration site is indicated by the yellow star, and other NYSDEC sites within the NYCMA are indicated by cyan triangles. (b) Rooftop at the NYSDEC Queens College calibration site, where sensor packages (yellow “*x*”) were colocated with reference-grade sensors before field site deployments (photo credit: Jason Covert). (c) Low-cost sensor package mounted to a NYSM tripod tower, approximately 2 m above the roof at the Bronx Van Nest (BXVNST) field site (photo credit: Lee Brittle). The map in 1a was created using QGIS^44^ with a basemap by Esri^45^ and U.S. states shapefile from the National Weather Service.^46^

The low-cost sensor packages were mounted to a custom wooden frame on the roof of the NYSDEC Queens College site trailer that accommodated up to 20 packages (Figure 1b, yellow *x*). The keystone package (AQA2020, described in Section 2.3) was colocated at the calibration site for 16 months (April 28, 2023 to August 31, 2024). A second long-term package (AQA2053) was deployed for 6 months (February 29, 2024 to August 31, 2024). Prior to deployment at field sites, each “field site package” was deployed at the calibration site for roughly 1 month. To match the resolution of the reference instruments, raw data from the packages were resampled from 5-s to hourly.

### 2.3 The Network Calibration Algorithm (NCA)

A schematic representation of NCA is shown in Figure 2. The basis of the NCA was the “keystone” package (Figure 2 red colors), which was permanently colocated with reference sensors at the calibration site. Synchronized data from the keystone package and reference sensors were used to train a single network calibration model (NCM) for each pollutant (upper box). The NCMs were subsequently applied to sensor packages deployed at the field sites (lower box). The remainder of this section details the pollutant-specific NCMs, the NCA performance for the keystone package, and how the NCMs developed for the keystone package were applied to the field packages.

**Figure 2:**
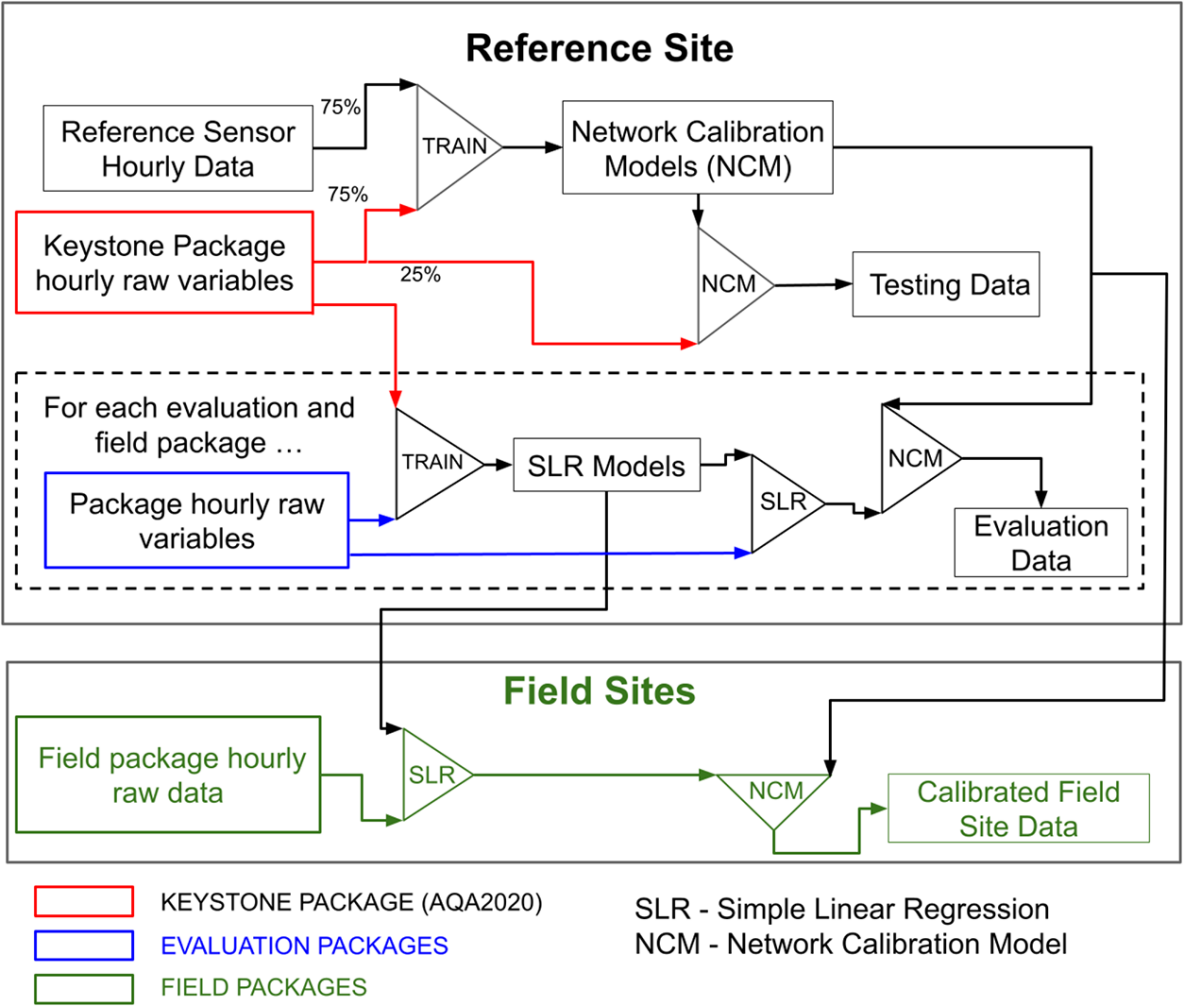
Schematic of the Network Calibration Algorithm (NCA) approach for low-cost sensor calibration. The NYSDEC Queens College monitoring site served as the reference site. Red colors correspond to the keystone package data stream; blue colors correspond to the field package data when deployed at the reference site, and green colors correspond to field package data when deployed at field sites.

#### 2.3.1 Pollutant-Specific Calibration Models

The relationships between low-cost sensor-measured variables and pollutant concentrations were calculated using two model types: multiple linear regression (MLR) and machine learning. MLR models have been widely used for low-cost sensor calibration due to their simplicity in application and interpretation.^5,12,22^ Machine learning models are able to resolve complex relationships between low-cost sensors and reference instrumentation by learning statistical relationships between variables, with the Random Forest (RF) model proven to be effective for calibration.^7,11,23^ The model type and predictors for each pollutant are given in Table 1. The simpler MLR model was used for CO and for the sum of the values of O_3_ and NO_2_. For CO, the predictor variables were simply the raw CO voltage (CO_RAW_) and its square (CO_RAW_)^2^. The squared term improved accuracy at low CO concentrations by reducing the frequency of negative predicted values, especially at rural sites where the background CO concentrations were expected to be lower than at the NYSDEC Queens College calibration site.^24^

**Table 1:** Network Calibration Model Configurations Per Pollutant^a^

**pollutant**	**MLR predictors**	**RF predictors**	**RF threshold** **(C_TH_)**
**PM**_**2.5**_	PM_2.5,RAW_, RH	PM_2.5,MLR_, PM_2.5,RAW_, RH, T	40 μg m^–3^
**O**_**3**_ **NO**_**2**_	O_3_NO_2,RAW_, T, RH	N/A	N/A
**NO**_**2**_	NO_2,RAW_, O_3_NO_2,RAW_, T, RH	NO_2,MLR_, NO_2,RAW_, O_3_NO_2,RAW_, O_3,RAW_ ^b^, CO_RAW_, NO_RAW_, T, RH	50 ppb
**O**_**3**_	O_3,EST_ ^c^, T, RH	O_3,MLR_, O_3,EST_ ^c^, T, RH	75 ppb
**NO**	NO_RAW_, NO_2,HYBRID_, T·NO_RAW_, T, RH	NO_MLR_, NO_RAW_, NO_2,HYBRID_,T·NO_RAW_, T, RH	40 ppb
**CO**	CO_RAW_, (CO_RAW_)^2^	N/A	N/A

a Predictors in the MLR models were statistically significant and chosen based on findings from similar studies and known cross-sensitivities.^5,7,10,12,18^ The MLR model coefficients are provided in the Supporting Information (Section S2).

b O_3_ RAW = O_3_NO_2_–NO_2_ (all uncalibrated).

c O_3_ EST = O_3_NO_2_ MLR–NO_2_ Hybrid.

The calibrations for PM_2.5_, O_3_, NO_2_, and NO required more complex models than MLR to mitigate the prediction of negative pollutant concentrations while preserving the ability of the MLR to extrapolate beyond the concentrations used in training.^7,10,23^ A “hybrid” model first applied a MLR to obtain an estimate of the pollutant that was assumed to be valid for concentrations above a threshold C_TH_. For concentrations below C_TH_, the MLR estimate and other predictors were used as inputs to an RF model (Table 1). The additional predictors were supplied to the RF models to capture more complex relationships, and the RF inherently disregarded predictors that were not found to be impactful. The RF thresholds listed in Table 1 were determined visually as the concentration below which the MLR-calibrated concentrations were not linearly related to the reference concentration (Figure S3). Other studies using hybrid MLR-RF models determined the thresholds statistically (e.g., Malings et al. 2019 set C_TH_ to 90% of the maximum training concentration); however, we found that the visual approach gave similar results. The RF models were applied using the Scikit Learn version 1.5.2 RandomForestRegressor function with 500 trees and a minimum of 10 samples required to split a node with nodes split using a criterion of absolute error. While in this work MLR and hybrid models were used, the NCA framework is flexible to use other model types (e.g., Gradient Boosting, Support Vector Regression, etc.).

The O_3_ and NO_2_ measurements were interdependent based on the output of both the Alphasense OX-B431 and NO2-B43F sensors. The OX-B431 (or “O_3_+NO_2_ sensor”) output the sum of O_3_ and NO_2_. The NO2-B43F used the same core sensor as the OX-B431; however, a built-in O_3_ “scrubber” removed O_3_ from the measurement volume such that, when functioning optimally, the NO2-B43F sensor reflected only the NO_2_ concentration.^25^ The O_3_ concentration was then determined as the difference between the corresponding outputs of the O_3_+NO_2_ and NO_2_ sensor outputs. The O_3_+NO_2_ sensor was calibrated using an MLR with temperature and RH as predictors and the sum of reference O_3_ and NO_2_ concentrations as the target variable (Table 1). The NO_2_ sensor was calibrated using a hybrid model with temperature, RH, and the O_3_+NO_2_ sensor as predictors for the MLR, and the output from all electrochemical sensors included in the RF model with an RF threshold of 50 ppb. Including output from all electrochemical sensors to calibrate NO_2_ using a RF model was used by Zimmerman et al. (2018)^7^ who found nontarget sensors like CO to improve performance. With NO_2_ calibrated, we derived a first estimate of O_3_ concentration by subtracting the hybrid-calibrated NO_2_ from the MLR-calibrated O_3_+NO_2_. This first estimate (“O_3_ EST” in Table 1) was used along with temperature and RH as inputs to a hybrid model for O_3_ with an RF threshold of 75 ppb.

#### 2.3.2 The NCA Training Data Set

The data set used to develop the calibration models spanned 16 months (May 2, 2023 to August 31, 2024), and was split into a training data set (75%) and a testing data set (25%, Figure 2). For each of the 16 months, three randomly chosen weeks of hourly raw data from the keystone package (AQA2020), along with the reference sensor concentrations, were included in the training data set. The remaining week from each month that was not included in the training data set was used to test model performance. The distribution of training and testing weeks is provided in Figure S4. Alternatives to the 75/25 training/testing split were tried to evaluate model sensitivity to the training data, including a more advanced 5-fold cross validation approach. Since these configurations had little impact on the computed pollutant concentrations, we chose to retain the conceptually simpler training/testing data split shown in Figure 2.

The hourly averaged meteorological conditions and pollutant concentrations included in the training and testing data are summarized in Figure 3. The solar radiation, air temperature, and humidity showed strong seasonal variation (Figure 3a–f), which, as discussed above, complicates the development of calibration models based upon *short-duration* colocations at the reference site. For the pollutants in the left column of Figure 3g,i,k,m,o, the reference concentrations are plotted in black, and the calibrated keystone package concentrations are shown in colors. O_3_ and NO showed the strongest seasonal cycles at the calibration site, while PM_2.5_ showed less of a seasonal cycle. NO_2_ and CO showed higher concentrations in the colder months.

**Figure 3:**
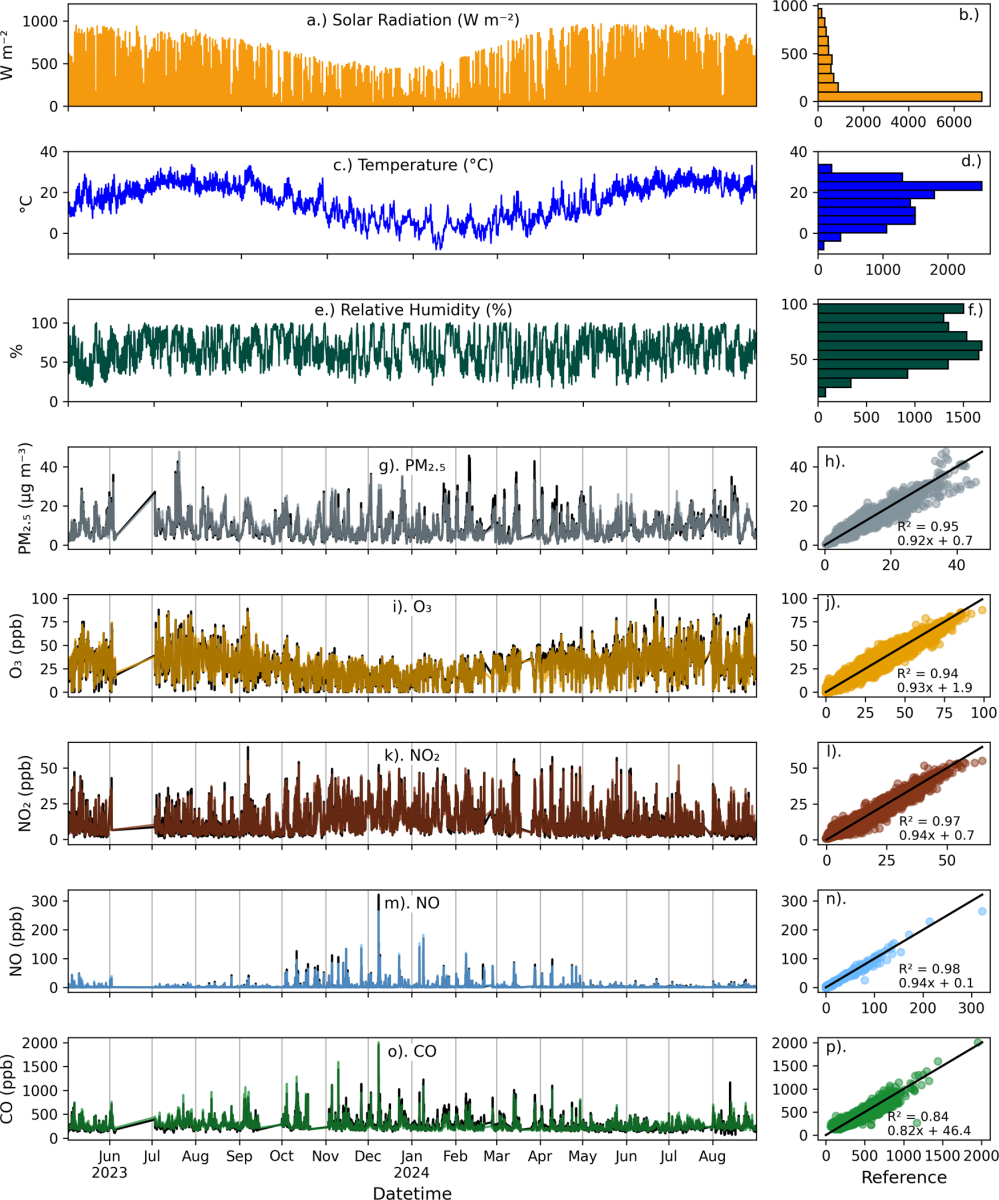
Hourly meteorological data time series (left column) and histograms (right column) at the NYSM Queens site at 25 m above ground level: (a, b) solar radiation (W m^–2^); (c, d) air temperature (°C); and (e, f) relative humidity (%). Hourly pollutant concentrations at the NYSDEC Queens College calibration site are shown on in the left column, with reference sensor data in black and the calibrated low-cost sensor keystone package (AQA2020) in colors: (g) PM_2.5_ (μg m^–3^, gray); (i) O_3_ (ppb, yellow); (k) NO_2_ (ppb, brown); (m) NO (ppb, blue); and (o) CO (ppb, green). Scatterplots for the pollutants are shown in the right column: (h) PM_2.5_ (μg m^–3^, gray); (j) O_3_ (ppb, yellow); (l) NO_2_ (ppb, brown); (n) NO (ppb, blue); and (p) CO (ppb, green), with a 1:1 line in black. The Queens NYSM site is located 0.57 km SW of the NYSDEC Queens College calibration site (Figures 1 and S2).

The ranges of pollutant concentrations measured by the reference monitors over the 16 months are summarized in Table S1. PM_2.5_ ranged from 0.3–411.1 μg m^–3^, with a median concentration of 13.7 μg m^–3^. PM_2.5_ values above ∼50 μg m^–3^ were due to several short, episodic events; specifically, the June 2023 Quebec, Canada wildfires and the July fourth fireworks in 2023 and 2024. The PM_2.5_ composition during these extreme events may differ from typical conditions and potentially bias the calibrations.^26–29^ Since trace gases such as CO and NO_2_ can also be affected by wildfire smoke and impact conditions needed to produce O_3_,^30,31^ data from June 5 to July 3 were flagged as the “wildfire period” and were omitted from the keystone package data set for all pollutants to ensure the NCA was trained using data representative of typical rather than extreme environmental conditions. Similarly, data from July 4, 15Z to July 5, 15Z were removed due to the influence of fireworks. These data omissions resulted in roughly 15 months of training data, with 15 weeks reserved for testing within those months (Figure S4). With the wildfire data removed, the maximum reference PM_2.5_ concentration in the training data set was 45.6 μg m^–3^ and the median concentration was 5.9 μg m^–3^. The concentrations of the other pollutants were less affected by the extreme events: O_3_ range 0.0–99.0 ppb (median 29.0 ppb); NO_2_ range 0.0–64.6 ppb (median 8.5 ppb); NO range 0.0–322.2 ppb (median 0.7 ppb); and CO range 62.0–1952.0 ppb (median 231.0 ppb).

#### 2.3.3 Evaluation Metrics

Statistical measures were used to quantify the performance of the NCA-calibrated low-cost sensors (Table 2). The coefficient of determination (*R* ^2^) measured the ability of the low-cost sensors to capture variability in the reference concentrations, with an *R* ^2^ of 1 indicating a perfect fit. Accuracy was quantified using root-mean-squared error (RMSE), mean absolute error (MAE), and mean bias error (MBE). A positive MBE indicates the calibrated sensors overestimated reference concentrations, and a negative MBE indicates reference concentrations were underestimated. Normalized estimates of the absolute error (NMAE) and bias error (NMBE) were calculated by dividing the MAE and MBE by the mean reference concentration.

**Table 2:** Performance of the NCA on Field Site Packages During Their Colocations at the NYSDEC Queens College Calibration Site^a^ ^,^ ^b^

	**Colocation** ^c^	**Mean Reference Concentration**	***R*** ^**2**^	**RMSE**	**MAE**	**NMAE**	**MBE**	**NMBE**
**PM**_**2.5**_ (μg m^–3^)	1	9.4	0.90 ± 0.02	1.8 ± 0.1	1.3 ± 0.1	0.14 ± 0.01	0.1 ± 0.2	0.01 ± 0.02
	wildfire	31.2	0.99 ± 0.00	18.2 ± 0.1	7.4 ± 0.0	0.24 ± 0.00	–6.7 ± 0.0	–0.22 ± 0.00
	2	9.0	0.91 ± 0.02	1.8 ± 0.2	1.2 ± 0.1	0.14 ± 0.01	–0.3 ± 0.3	–0.04 ± 0.03
**O**_**3**_ (ppb)	1	35.2	0.83 ± 0.02	6.7 ± 0.3	5.2 ± 0.3	0.15 ± 0.01	–1.0 ± 0.6	–0.03 ± 0.02
	2	29.1	0.85 ± 0.03	5.7 ± 0.7	4.3 ± 0.5	0.15 ± 0.01	–1.3 ± 0.9	–0.04 ± 0.03
**NO**_**2**_ (ppb)	1	11.0	0.82 ± 0.03	3.9 ± 0.2	2.8 ± 0.2	0.26 ± 0.02	–0.4 ± 0.3	–0.03 ± 0.03
	2	11.9	0.82 ± 0.05	4.1 ± 0.5	2.9 ± 0.4	0.28 ± 0.07	1.2 ± 0.6	0.14 ± 0.07
**NO** (ppb)	1	2.0	0.83 ± 0.03	2.1 ± 0.2	1.2 ± 0.1	0.64 ± 0.07	0.7 ± 0.2	0.37 ± 0.07
	2	3.4	0.77 ± 0.11	2.3 ± 0.5	1.1 ± 0.2	0.43 ± 0.09	–0.3 ± 0.2	–0.07 ± 0.04
**CO** (ppb)	1	262.8	0.90 ± 0.02	44.0 ± 2.7	33.8 ± 2.4	0.13 ± 0.01	16.0 ± 5.7	0.06 ± 0.02
	2	258.0	0.80 ± 0.08	52.0 ± 4.1	38.2 ± 2.9	0.15 ± 0.01	–5.7 ± 12.0	–0.01 ± 0.05

a For each colocation period, statistics are first calculated for each sensor package. The package-specific values are then used to calculate averages and 95% confidence intervals.

b Data from reference monitors operated by the NYSDEC were used for evaluation.

c Colocation 1 refers to 57 field packages deployed at the reference site prior to their first field deployment. Colocation 2 refers to 25 field packages deployed at the reference site after field deployment. For PM_2.5_, data from 18 packages from June 6, 2023, to July 3, 2023, are identified as “wildfire” packages due to their exposure to the Quebec, Canada wildfires. Around 50,000 h are included in colocation 1, with about 11,200 h in the wildfire subset. Colocation 2 is composed of over 26,000 h of data.

#### 2.3.4 NCA Testing on Keystone Package

The hourly calibrated concentrations from the keystone package are shown along with reference concentrations in Figure 3g–p. For all pollutants, the NCMs reproduced seasonal patterns and captured both low and high peaks. During testing, the calibrated PM_2.5_, O_3_, NO_2_, and NO concentrations were well correlated with the reference concentrations (*R* ^2^ > = 0.89), while the CO *R* ^2^ was slightly lower (0.84). The NCA performance statistics for the keystone package during training and testing periods are listed in Table S2. The MAE was 1.2 μg m^–3^ for PM_2.5_, 4.0 ppb for O_3_, 2.1 ppb for NO_2_, 0.5 ppb for NO, and 40.9 ppb for CO. Upon normalizing the MAE using the mean reference concentrations, the NMAE averaged near 15% for PM_2.5_, O_3_, NO_2_, and CO and 19% for NO. The magnitude of the NMBE for all pollutants was 4% or less.

#### 2.3.5 Application of the NCA to Field Site Packages

The NCMs were computed using the keystone package data. To apply the NCMs to the other sensor packages, they were each colocated alongside the keystone package for roughly 1 month. The colocated data were used to compute simple linear regressions (SLRs or “transfer functions”) that mapped each raw variable in each package to the corresponding raw variable in the keystone package (Figure S5). Obtaining calibrated values for field packages was then a two-step process, where the raw data were first mapped to the keystone package using their unique SLRs and then fed into the NCMs (Figure 2, dashed box, green shapes, and text). Plotting the MBE as a function of days used to train the SLRs (Figure S6) indicates that stable SLRs can be obtained by training on relatively short periods; SLRs for NO_2_, O_3_, and NO stabilized after 1–5 days, while the PM_2.5_ and CO sensors required closer to 20 days. Additional information regarding the SLRs, including performance validation using the keystone package (Table S3), can be found in the Supporting Information (Section S3; Tables S3 and S4, Figures S5 and S6).

### 2.4 Field and Calibration Site Deployments

Of the 60 low-cost sensor packages that were fabricated, 2 remained at the NYSDEC Queens College calibration site (the keystone package and the evaluation package) and 1 remained at UAlbany for testing and development; the remaining 57 packages were used for field site deployments. Each field package was first colocated at the calibration site for ∼1 month to develop the SLR transfer functions that mapped their raw signals to the keystone package (black dashed box in Figure 2). A schedule of package deployments to the NYSDEC Queens College calibration site is shown in Figure S7. The age of the packages, quantified as days since initial deployment, is shown in Figure S8.

Following initial colocation at the calibration site, packages were deployed to field sites. At NYSM standard sites (Figure 1a, yellow circles), packages were installed on 10-m tall towers at a height of 2 m, while at the urban micronet sites, packages were mounted to tripod towers (Figure 1c). The network of 38 field sites covers a broad range of environmental settings, including urban, suburban, and rural locations. Information about the field sites can be found in Miller et al. (2025).^20^ The 19 sensor packages that were not deployed at the calibration or field sites or reserved for lab testing provided a cache of calibrated packages for seamless replacement at field sites, ensuring data continuity and minimizing downtime. During the course of the project from April 8, 2023 to August 31, 2024, packages were removed from some field sites and returned to the calibration site (Figure S7, red markers). These 25 additional colocations were used to recheck the initial SLR transfer functions and to assess stability of the calibration models.

## 3 Results and Discussion

### 3.1 Evaluation Data Sets

The performance of the NCA when applied to packages other than the keystone package was quantified using several independent “evaluation” data sets: (1) a long-term “evaluation package” (AQA2053) deployed at the calibration site for over 6 months; (2) field packages when initially deployed at the calibration site prior to their first field deployment (“colocation 1”); (3) field packages upon returning from field sites to the calibration site (“colocation 2”); and (4) field site packages compared to nearby, though not colocated, NYSDEC reference monitoring sites within the NYCMA (referred to as “nearby field evaluations”). The colocation 1 data were primarily used to assess the accuracy of the calibrated sensors, while the colocation 2 and long-term evaluation package data were used to assess stability of the NCA-calibrated low-cost sensors. The nearby field evaluations provided insight into the performance of the NCA in a range of environments, some very different from the NYSDEC Queens College calibration site.

The NCA performance during colocations 1 and 2 is summarized in Table 2. Data from 57 field packages were included in colocation 1. These packages were deployed in groups of 11–19, with a median deployment period of 55 days (range 20–159 days), totaling over 50,000 h. The colocation 2 data set consisted of 25 packages that were removed from field sites and returned to the calibration site for a median duration of 63 days (range 17–163 days), totaling over 24,000 h. The average package age at the start of colocation 2 was 293 days (range 98–424 days). The data during the June 2023 Quebec, Canada wildfires were excluded from the colocation 1 data set and, for PM_2.5_, comprised a separate “wildfire” category that was used to assess the performance of the NCMs for PM_2.5_ during wildfire conditions (Table 2). Colocation 1 and Colocation 2 data are aggregated together in Table 3 and compared to similar studies, with the wildfire PM_2.5_ data omitted.

**Table 3:** Comparison with Similar Work for the NCA Validated at the NYSDEC Queens College Calibration Site, a More Comprehensive Table for Each Pollutant Can Be Found in Table S7–S11 ^a^

**Pollutant**	**Study**	**Mean Reference Concentration**	***R*** ^**2**^	**MAE**	**MBE**
**PM**_**2.5**_	**Hojeily et al. 2025** ^b^	**9.3 ± 0.3 μg m** ^**–3**^	**0.90 ± 0.01**	**1.3 ± 0.1 μg m** ^**–3**^	**–0.0 ± 0.2 μg m** ^**–3**^
	Barkjohn et al. 2021 ^18^ ^,^ ^c^	4–10 μg m^–3^	0.83	1.8 μg m^–3^	0 μg m^–3^
	Raheja et al. 2023 ^33^ ^,^ ^d^	5.2–60 μg m^–3^	0.89	1.9 μg m^–3^	NR
**O**_**3**_	**Hojeily et al. 2025**	**33.5 ± 1.2 ppb**	**0.84 ± 0.02**	**4.9 ± 0.3 ppb**	**–1.1 ± 0.5 ppb**
	W25^e^	26.8 ppb	0.95 (0.88)	1.9 (2.9) ppb	–0.3 (0.0) ppb
	M19^f^	22–48 ppb	0.73 ± 0.12	5.9 ± 1.5 ppb	0.9 ± 2.9 ppb
**NO**_**2**_	**Hojeily et al. 2025**	**11.1 ± 0.5 ppb**	**0.82 ± 0.02**	**2.8 ± 0.2 ppb**	**0.1 ± 0.3 ppb**
	W25	6.4 ppb	0.82 (0.66)	1.7 (2.4) ppb	0.3 (0.9) ppb
	M19	4–9 ppb	0.30 ± 0.17	3.4 ± 0.5 ppb	0.2 ± 2.6 ppb
**NO**	**Hojeily et al. 2025**	**2.4 ± 0.4 ppb**	**0.81 ± 0.04**	**1.2 ± 0.1 ppb**	**0.4 ± 0.2 ppb**
	W25	2.4 ppb	0.82 (0.79)	1.0 (1.1) ppb	0.3 (0.4) ppb
	M19	1–3 ppb	0.17 ± 0.08	13.2 ± 22.7 ppb	9.9 ± 20.1 ppb
**CO**	**Hojeily et al. 2025**	**259.9 ± 6.7 ppb**	**0.87 ± 0.03**	**35.2 ± 1.9 ppb**	**9.4 ± 5.7 ppb**
	W25	158.0 ppb	0.90	20.2 ppb	–1.5 ppb
	M19	145–451 ppb	0.85 ± 0.09	56.0 ± 8.0 ppb	6.0 ± 93 ppb

a M19 = Malings et al. (2019)^10^, W25 = Winter et al. (2025)^12^, NR = Not reported.

b A total of 82 colocation data sets (colocations 1 and 2 combined) were used with the wildfire data (June 5 to 3 July 3, 2023) removed from PM_2.5_.

c Statistics are taken from Barkjohn et al. (2021)^18^ Table S8 for their “2+RH” correction model evaluated using a leave out by date validation.

d Statistics are taken from Raheja et al. (2023)^33^ Table 3 from their PurpleAir RF model, additional statistics for their other models (i.e., MLR, XGBoost) are provided in Table S7.

e Statistics are taken from Winter et al. (2025)^12^ for their colocated (remote) calibration models, except for CO where only the colocated model is reported.

f Statistics are taken from Malings et al. (2019)^10^ Table S4 for their general hybrid “HY” model for all pollutants but CO where we use their ‘LQR’ model, and reference range from their Table 4. Results are presented as the average ± the standard deviation for M19.

### 3.2 Performance of NCA-calibrated Low-cost Sensors

#### 3.2.1 Coefficient of Determination, *R* ^2^

Time series of all pollutants during a 16-day period (May 2, 2023 to May 19, 2023) when 8 packages were deployed at the calibration site are shown in Figure 4. The calibrated sensors show remarkable agreement with the reference concentrations for all pollutants at hourly and daily time scales, with little spread among the 8 sensor packages (Figure 4, left column). During this period, the low-cost sensors were highly correlated (*R* ^2^ > 0.90) with the reference for all pollutants (Figure 4, right column). The data shown in Figure 4 amounted to roughly 10% of the colocation 1 data set; statistics for the full data set are presented in Table 2. For the entire colocation 1 data set, the *R* ^2^ values for the 57 packages were somewhat lower than shown in Figure 4, ranging from 0.82 for NO_2_ to 0.90 for CO. The *R* ^2^ values across the network during colocation 1 were comparable to performance on the keystone and evaluation packages during testing.

**Figure 4:**
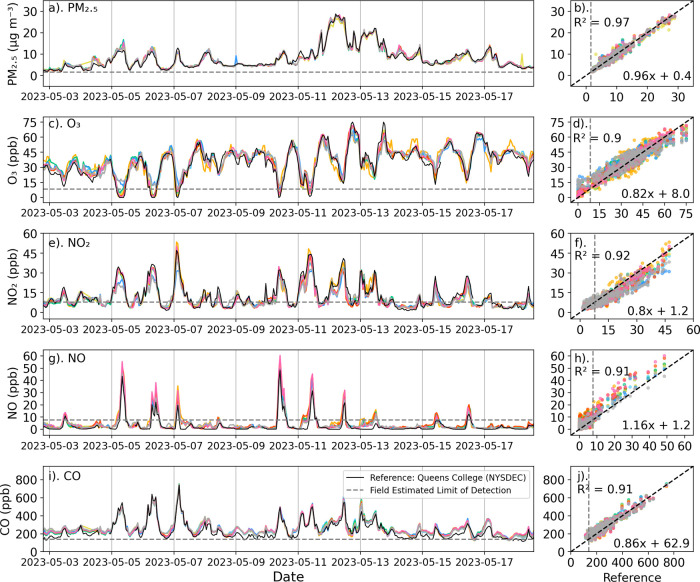
Hourly network-calibrated low-cost sensor pollutant concentration time series (left column) from 8 sensor packages (colored curves) along with reference sensor concentrations (black curves) during colocation at the NYSDEC Queens College calibration site from May 2, 2023–May 19, 2023: (a) PM_2.5_ (μg m^–3^); (c) O_3_ (ppb); (e) NO_2_ (ppb); (g) NO (ppb); and (i) CO (ppb). The right column shows scatterplots of the hourly low-cost sensors (y-axis) versus the reference observations (x-axis): (b) PM_2.5_ (μg m^–3^); (d) O_3_ (ppb); (f) NO_2_ (ppb); (h) NO (ppb); and (j) CO (ppb). The 1:1 line is shown in black, and the average *R* ^2^ and linear regression slope and offset are indicated in the scatterplots. The horizontal dashed lines are the fLODs (Section 3.2.4).

#### 3.2.2 Accuracy

The accuracy of the NCA-calibrated sensors was quantified using MAE and MBE from the colocation 1 data set (Table 2). The PM_2.5_ MAE was 1.3 μg m^–3^, similar to other studies that used the same sensor.^18,32,33^ When normalized by the colocation 1 mean PM_2.5_ concentration of 9.4 μg m^–3^, the NMAE for PM_2.5_ was roughly 14%. We note that the PM_2.5_ NCM was trained using only non-wildfire data. When this NCM was applied to packages deployed at the calibration site *during the wildfire period*, PM_2.5_ concentrations measured by the reference sensors reached over 350 μg m^–3^, and the MAE increased 5-fold to 7.4 μg m^–3^. The increased MAE presumably reflected the sensitivity of the PMS5003 sensor to differences in PM_2.5_ composition between wildfire and non-wildfire aerosol.^26^

The accuracy of the NCMs for gaseous pollutants were compared to other low-cost sensor networks in Pittsburgh (Malings et al., 2019; hereafter “M19”)^10^ and San Francisco (Winter et al., 2025; hereafter “W25”).^12^ Numerous types of calibration models were evaluated in M19; here, we compare performance statistics with their general hybrid models produced for O_3_, NO_2_, and NO and their general quadratic MLR model for CO. In this study, the MAE for O_3_ was 5.2 ppb, similar to M19 and roughly a factor of 3 higher than W25, presumably due to higher O_3_ concentrations in Pittsburgh and NYC compared to San Francisco. The MAE for NO_2_ was 2.8 ppb, consistent with those of M19 and W25. The MAE for NO was 1.2 ppb, similar to W25 and much lower than 13.2 ppb estimated by M19. For CO, the MAE was 33.8 ppb, between the 20.2 ppb of W25 and the 56 ppb of M19. When normalized by their mean concentrations, the gas sensor NMAEs ranged from 13% for CO, 15% for O_3_, 26% for NO_2_, and 64% for NO. The higher NMAE for NO was driven by its low mean concentration (2.0 ppb).

To evaluate environmental sensitivities, the low-cost sensor errors are plotted against RH and temperature (Figure S9), month (Figure S10), and reference concentration (Figure S11). The PM_2.5_, NO_2_, and NO did not show significant biases (below 25% or above 75% quartile ranges) for any of the RH bins, while O_3_ showed a small negative bias (median roughly −2.5 ppb) for the lowest RH bin 20% and CO showed small positive biases (median roughly 25 ppb) for RH bins between 60–90% (Figure S9a). Several pollutants showed small temperature-correlated biases: PM_2.5_ median error ∼1 ug m^–3^ for temperatures between 35–40 C; O_3_ median bias roughly −2.5 ppb at temperatures less than 5 °C; NO_2_ median bias roughly 3 ppb at temperatures less than 0 C; and CO median error roughly −25 ppb for temperatures 0–5 C and 30 ppb for temperatures 20–30 °C (Figure S9b). For NO, O_3_, and CO, small positive biases were found during the warmer seasons and small negative biases during the colder seasons (Figure S10). The error for PM_2.5_ was notably negative in June 2023, presumably due to underestimations related to the wildfire smoke. NO_2_ developed a positive bias starting in late spring 2024, which may have been caused by sensor aging, as described in Section 3.2.3.

The pollutant concentration errors are plotted in Figure S11 as a function of the range of reference concentrations of each pollutant observed during the colocations. We note that these plots may be indicative of correlation rather than causation, as some errors may be driven by similar meteorological conditions rather than a genuine cross-sensitivity. For example, the O_3_ error was biased negative at higher concentrations of both reference PM_2.5_ and O_3_ (Figure S11a,b). Generally, Figure S11 shows the calibrated pollutant errors to be stable across the range of reference concentrations, with some exceptions: NO was underestimated by 5 to 15 ppb at high concentrations of NO_2_ (>50 ppb), and CO was overestimated by 50–200 ppb at CO concentrations above 700 ppb (Figure S11e).

The accuracy of the NCMs generally improved with the length and seasonality of the training period. To demonstrate this, the NCMs were recomputed for training period lengths ranging from 3 to 72 weeks (Figure 5), with all periods having the same start date (May 2, 2023). For each training period length, the MBE for each pollutant was calculated from the testing data set (i.e., hourly data for one randomly selected week for each month over the 15-month training data set, Figure S4). The left-most points in each panel of Figure 5 correspond to training the NCMs on short calibration periods and then applying those calibrations to the 15-month testing data set, similar to the typical calibration approach (i.e., short-term colocations). As the number of training period weeks increased from 3 to 10, the MBE for all pollutants changed markedly, reinforcing the uncertainty associated with short calibration periods. As the training period length increased from 10 to 25 weeks, the MBE magnitudes for PM_2.5_, O_3_, and NO_2_ decreased while those for NO and CO remained small and roughly constant at −1 and −30 ppb, respectively. Beginning in October (25 weeks), ambient temperatures cooled, and MBE magnitudes for O_3_, NO, and CO decreased, highlighting the importance of including a broad range of meteorological conditions in the training data. The MBE magnitudes approached their minimums after 40–50 weeks for NO, 50–60 weeks for PM_2.5_, O_3_, and NO_2_, and 70 weeks for CO. Our results are consistent with Levy Zamora et al. (2023)^34^ and demonstrate that for NYC, which has strong meteorological and pollutant seasonality, longer colocations are beneficial for calibration models by providing them with representative training data.

**Figure 5:**
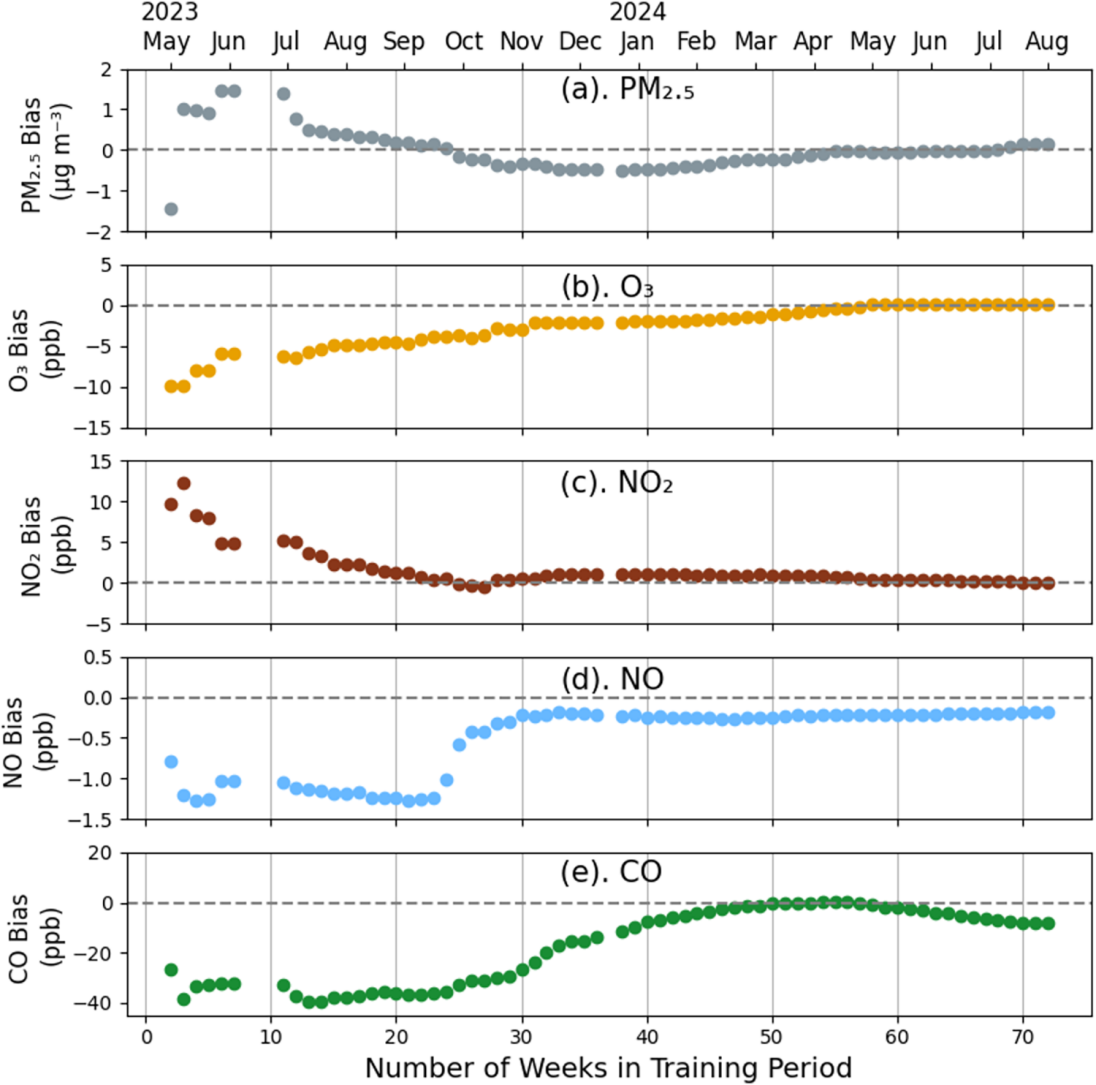
Mean bias error (MBE) versus number of weeks included in the training period for the Network Calibration Models: (a) CO (ppb); (b) PM_2.5_ (μg m^–3^); (c) O_3_ (ppb); (d) NO_2_ (ppb); and (e) NO (ppb). For all points, the training period began on May 2, 2023. The NCMs were calculated using the keystone package training data (AQA2020), and the MBE was calculated using the testing data (1 week per month, Figure S4).

#### 3.2.3 Stability

A key challenge for long-term (>1 year) deployment of low-cost sensors is that their performance can change over time. Extrapolating calibrations based on short-term (e.g., 1-month) colocation periods at a reference site (the typical approach) across seasons with different meteorological and pollutant conditions can result in prediction errors.^34–37^ In addition, the electrochemical sensors degrade over time due to depletion of their aqueous electrolyte solution (“sensor drift”); their nominal usable lifetime is manufacturer-specified to be roughly 2 years.^38^ Further, the ability of the NO2-B43F sensor to solely measure NO_2_ degrades as the built-in O_3_ scrubber is consumed, causing the sensor to increasingly respond to both NO_2_ and O_3_.^25,38^ The manufacturer specifications indicate that the O_3_ scrubber is depleted after 250 ppm-days of exposure to O_3_, which, based on the average O_3_ concentration of 28 ppb at the NYSDEC Queens College calibration site in 2023, corresponds to roughly 400 days. While some studies have addressed drift by using time as an explicit predictor,^5^ here the RF component of the hybrid models accounted for time implicitly as the relationships between the predictor variables changed seasonally and as the sensors degraded.

Stability of the NCA-calibrated sensors over a continuous 6-month period was assessed using the long-term evaluation package deployed at the calibration site (AQA2053). Time series are plotted in Figure 6, and performance statistics are tabulated in Table S2 along with statistics for the keystone package. Similar to the keystone package (Figure 3), the hourly time series from the evaluation package showed close agreement with the reference sensor concentrations, matching both low and high values (Figure 6, left column). Beginning around May 2024, the O_3_ values appeared to somewhat underestimate the reference concentrations (Figure 6c), while the NO_2_ values appeared to overestimate the reference concentrations (Figure 6e). The signs of these changes in the O_3_ and NO_2_, compared with the reference sensors, were consistent with the onset of the O_3_ scrubber depletion effects on the NO2-B43F sensor. However, the magnitude of these differences was much smaller using the NCA compared to using typical individual calibration models based upon short-duration colocations.^39^

**Figure 6:**
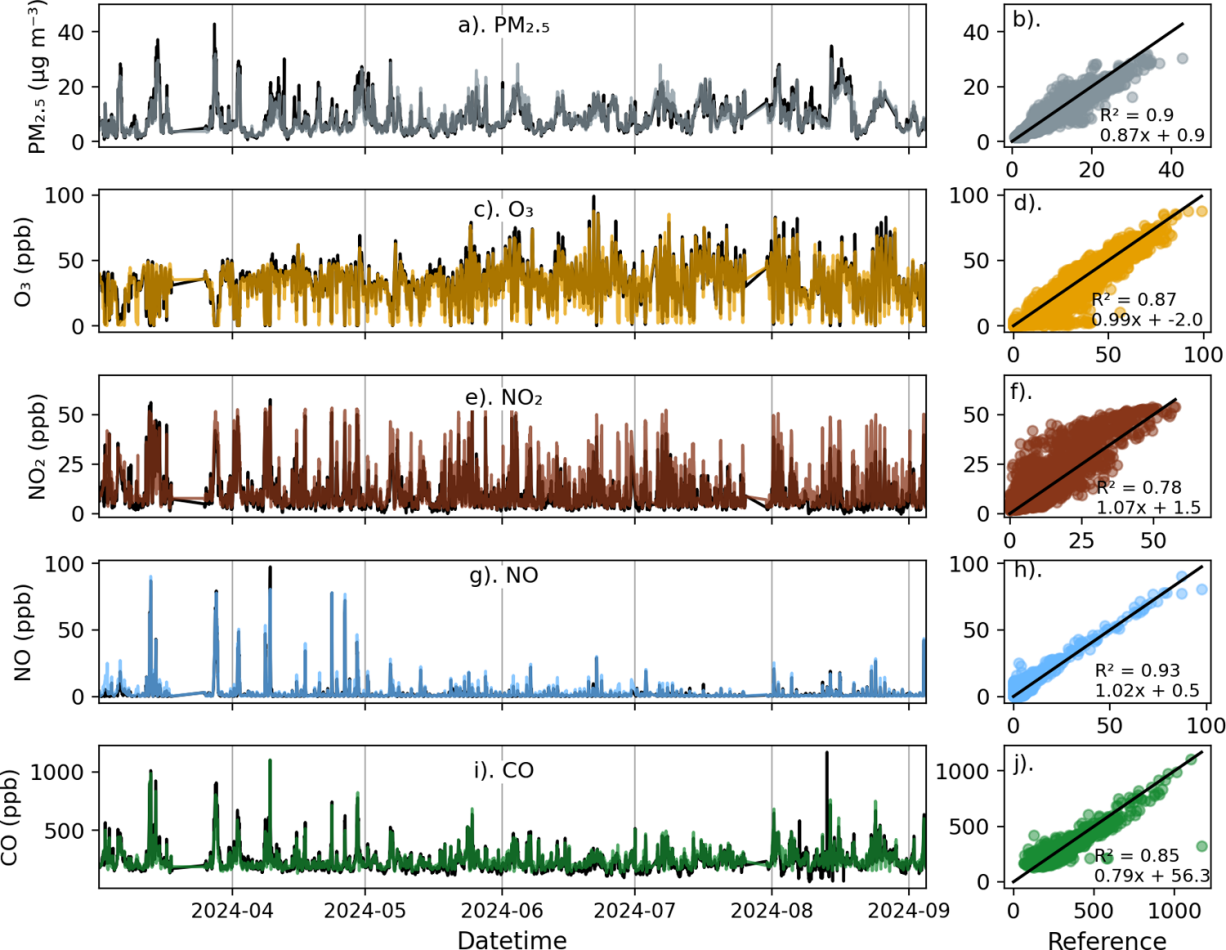
Six months of hourly pollutant concentrations at the NYSDEC Queens College calibration site measured by the network-calibrated low-cost sensor evaluation package (AQA2053, colored curves), along with reference sensor data (black curves) between March 1, 2024 and August 31, 2024: (a) PM_2.5_ (μg m^–3^, gray); (c) O_3_ (ppb, yellow); (e) NO_2_ (ppb, brown); (g) NO (ppb, blue); and (i) CO (ppb, green). Scatterplots for the pollutants are shown in the right column: (b) PM_2.5_ (μg m^–3^, gray); (d) O_3_ (ppb, yellow); (f) NO_2_ (ppb, brown); (h) NO (ppb, blue); and (j) CO (ppb, green), with a 1:1 line in black.

Data from colocation 2, when 25 packages were returned to the calibration site after field deployment, were used to quantify calibration performance for packages with ages ranging from 98 to 424 days, providing insight into the stability of the NCA-calibrated sensors. The first test involved recalculating the SLR transfer functions for colocation 2 and comparing computed pollutant concentrations using colocation 1 SLRs with those computed using colocation 2 SLRs (Table S4). For PM_2.5_, NO_2_, NO, and CO, the changes in the *R* ^2^ values, MAE, and MBE using colocation 1 versus colocation 2 SLRs were not significant at the 95% confidence level, while changes in the MAE for O_3_ were barely significant.

The NCA-calibrated sensor stability was also assessed by comparing the performance statistics between colocation 1 and colocation 2 (Table 2). Small changes in *R* ^2^, RMSE, and MAE between colocation 1 and 2 were found for all pollutants; however, most were not significant at the 95% confidence level. Exceptions were for O_3_ which had significantly lower (i.e., improved) RMSE (6.7 to 5.7 ppb) and MAE (5.2 to 4.3 ppb) for colocation 2. For NO_2_, the MBE was 1.6 ppb higher during colocation 2. Both NO and CO showed statistically significant decreases in MBE from colocation 1 to colocation 2: NO from 0.7 to −0.3 ppb and CO from 16.0 to −5.7 ppb.

The colocation 2 NMBEs are plotted against package age in Figure 7, with a cluster of package ages between 100–250 days and another cluster for ages longer than 325 days, coinciding with batch deployments. The NMBE for PM_2.5_, O_3_, and NO were not strongly dependent upon package age for the range of colocation 2 ages, though the range of NMBE for NO appears larger for package ages above 300 days than for younger packages, indicating more variability in performance with increasing age (Figure 7, blue circles). For CO, packages less than 250 days old showed a negative NMBE (underestimate) of around −0.1, which shifted to an overestimate (positive NMBE) of roughly the same amount for package ages above 300 days. The strongest dependence on package age was for the NO_2_. For ages less than 300 days, the NMBE was close to zero. For package ages above 300 days, most of the packages overestimated NO_2_ by up to ∼50% (the average NO_2_ overestimate was 14%, Table 2). This positive bias was consistent with the O_3_ scrubber depletion in the NO2-B43F sensor. Interestingly, the NMBE for O_3_ was consistent across package age despite its dependency on the NO_2_.

**Figure 7:**
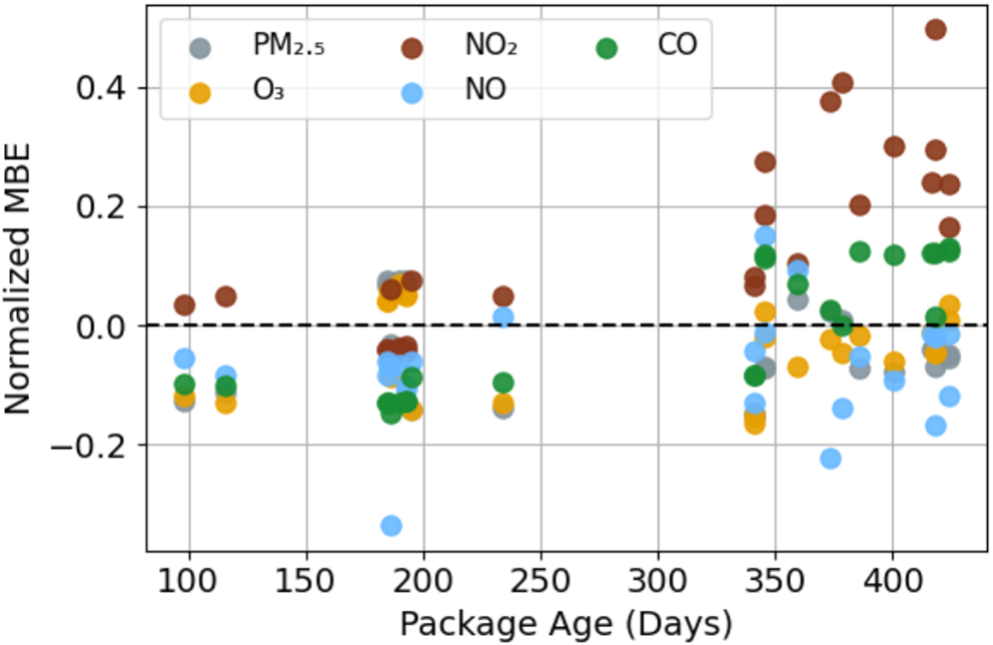
NMBE during colocation 2 at the NYSDEC Queens College site (*y*-axis) as a function of a package age at the start of colocation 2 (*x*-axis). Data for the 25 packages are plotted.

Hourly differences between pollutant concentrations measured at the NYSM Queens site and the nearby reference concentrations at the NYSDEC Queens College calibration site were used to compute weekly MBEs that reveal temporal trends in the performance of NCA (Figure 8). Note that these two sites were not colocated, but rather separated by 570 m (Figure S2). The MBEs for PM_2.5_ and NO were close to zero for most of the 6-month period, with slightly negative PM_2.5_ biases during spring and late summer. The CO MBE suggests a seasonal or temperature bias, with a negative MBE in early spring and a positive MBE in summer. After roughly 8 months, O_3_ was underestimated by the NCA while NO_2_ was overestimated - these changes may reflect residual O_3_ scrubber depletion effects that were not accounted for by the NCA.

**Figure 8:**
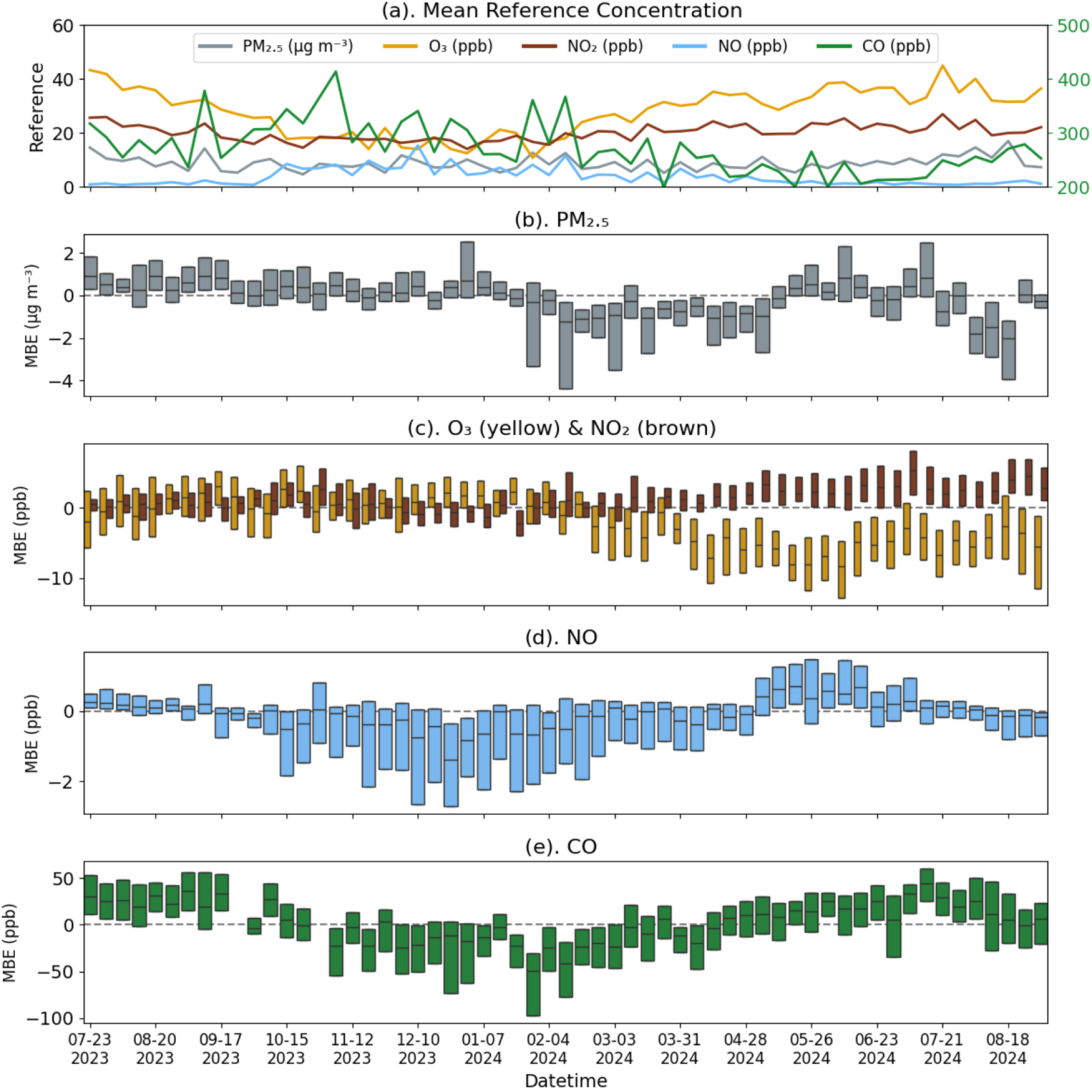
Weekly boxplots of MBE, calculated from the hourly difference between the NYSM Queens site concentrations and the NYSDEC Queens College calibration site. The two sites are separated by 570 m (Figure S2) (a) mean pollutant reference concentrations, (b) PM_2.5_ (μg m^–3^, gray); (c) O_3_ (ppb, yellow) and NO_2_ (ppb, brown); (d) NO (ppb, blue); and (e) CO (ppb, green, secondary *y*-axis (a)). Hourly time-series data for all pollutants are provided in Figure S12.

#### 3.2.4 Field Limit of Detection (fLOD)

The limit of detection (LOD) is defined as the lowest concentration that can be distinguished from measurement noise with a specified degree of certainty (e.g., 95%).^13^ Typically, the LOD is determined in a controlled laboratory setting by exposing sensors to air without the target species (“blanks”). Since laboratory testing was not part of this study, estimates of the “field LOD” (fLOD) were computed from hourly periods during colocation 1 and colocation 2 when the reference instrument ambient air concentrations were zero or below a threshold value (“pseudo blanks”, Table 4) according to 3σ_(*x*–xref)_/slope where σ is the standard deviation, *x*–xref is the difference between the low-cost sensor and reference instrument pollutant concentration, and “slope” is the linear regression slope of the low-cost sensor versus the reference instrument.^29^ The number of hourly pseudo blanks ranged from 105 for CO (threshold concentration 100 ppb) to 1450 for O_3_ (no threshold applied). Since the calculated fLODs showed slight dependence on the random weeks within each month that were used as the keystone package training data, a bootstrapping technique was used to quantify the sensitivity. The NCMs were computed 10 separate times, each with a different randomly selected training data set, and the average fLODs for the 10 replicates were computed and reported in Table 4, along with the range of fLODs for the 10 replicates.

**Table 4:** Field Limit of Detection (fLOD) Determined from Pseudo Blanks Using Hourly Data from Packages during Colocation at the NYSDEC Queens College Calibration Site^a^

	**PM**_**2.5**_ **(μg m** ^**–3**^ **)**	**O**_**3**_ **(ppb)**	**NO**_**2**_ **(ppb)**	**NO** **(ppb)**	**CO** **(ppb)**
pseudo blank threshold	<1.0	0.0	<1.0	0.0	<100
N pseudo blanks	161	1450	175	957	105
estimated fLOD	1.6	8.5	7.8	7.7	139.2
fLOD range	1.4–1.7	7.9–9.3	7.4–9.2	7.0–8.5	132.3–145.1
precision	5%	11%	12%	27%	3%
bias	–4%	–3%	–2%	23%	3%
EPA monitoring tier	Tier III	Tier III	Tier III	Tier II/IV	Tier III
field site mean ± 95% CI	9.1 ± 0.03	27.2 ± 0.04	11.3 ± 0.03	3.5 ± 0.03	247.0 ± 0.4
field site range	0.5–405.6	0.0–87.6	1.1–71.6	0.1–360.2	82.5–2498.5
% field observations greater than fLOD	99%	91%	49%	10%	97%

a Data from all calibrations used for calculations.

The estimated fLOD for PM_2.5_ was 1.6 μg m^–3^, consistent with other studies^29,32^ and much lower than the mean PM_2.5_ concentrations across the network of field sites (9.0 μg m^–3^). Ninety-nine percent of PM_2.5_ observations at the field sites were larger than the fLOD. The fLOD for O_3_ (8.5 ppb), NO_2_ (7.8 ppb), and NO (7.7 ppb) were similar; however, their field site concentrations were very different, and the percentage of field observations greater than the fLOD were 91% for O_3_, 49% for NO_2_, and 10% for NO. The calculated fLOD for CO was 139.2 ppb, which may represent an overestimate since concentrations at the calibration site (and thus the pseudo blank threshold) were high. Nonetheless, 97% of field site CO measurements were above the fLOD. For NO, we note that the calculated fLOD was sensitive to the ambient NO_2_ concentration; when the data used to compute the NO fLOD were filtered to include only periods when NO_2_ concentrations were low, estimates of the NO fLOD were closer to 4 ppb (compared with 7.7 ppb). The dependence of fLODs upon ambient conditions and pollutant concentrations may limit their utility as a stand-alone low-cost sensor performance metric.

### 3.3 Comparison with Nearby Reference Monitors

The NYSDEC maintains regulatory-grade monitoring sites in the NYCMA that measure one or more pollutants included in the low-cost sensor package (Figure 1a, cyan triangles, and Table S5). The data from these NYSDEC sites can be compared with *nearby* low-cost sensor air quality measurements at NYSM field sites; we refer to these comparisons as “nearby field evaluations” to distinguish from evaluations completed using data from packages colocated at the calibration site. The nearby data provide additional assessments of low-cost sensor accuracy and stability, though caution is warranted as paired sites are not colocated and the area surrounding site locations can vary spatially, and local sources may be different (e.g., proximity to roads).

Hourly time series of O_3_, NO_2_, NO, and CO were plotted for 3 nearby field evaluation site pairs: (1) NYSDEC Queens College and NYSM Queens (urban, 0.57 km apart, 12 month duration, Figures S2 and S12); (2) NYSDEC Pfizer Lab and NYSM Bronx (urban, 1.4 km apart, 11 month duration, Figures S13 and S14); and (3) NYSDEC Flax Pond and NYSM Stony Brook (suburban, 4.71 km apart, 6-month duration, Figures S15 and S16). Differences in the immediate areas surrounding the paired sites are highlighted in the captions for Figures S2, S13, and S15. The time series for each set of paired sites generally tracked one another closely for all pollutants for the entire nearby field evaluation period, with the NCA-calibrated low-cost sensors capturing the long-term (i.e., seasonal) trends as well as most of the short-duration peaks and spikes (Figures S12, S14, and S16). Notable differences were found; for example, the nearby low-cost sensor at the NYSM Queens site underestimated the magnitude of the highest NO peaks, while the *in situ* evaluation package at the NYSDEC Queens College site accurately reproduced the reference sensor peak magnitudes (Figure 6g). The underestimate of the peaks at the NYSM site may have been due to spatial variations between the two sites, which were separated by 570 m.

Statistical comparisons between hourly values from the reference instruments at the NYSDEC sites and NCA-calibrated low-cost sensors at nearby NYSM field sites are tabulated for 6 site pairs in Table S6. The distance between the NYSDEC reference sites and the nearest NYSM station with a low-cost sensor package ranged from 0.57 km (NYSM Queens to NYSDEC Queens College) to 13.9 km (NYSM Dover Plains to NYSDEC Millbrook). The site pairings showed moderate-to-high correlations (*R* ^2^ 0.49–0.89) across all pollutants (except NO), and correlations generally decreased with increasing distance between the paired sites (Table S6). The highest correlations were observed for O_3_, which tends to have a more homogeneous regional distribution.^24^ The lowest correlations were generally for NO, with the NYSDEC Flax Pond and NYSM Stony Brook site pair registering the lowest *R* ^2^ value (0.06). For NO_2_, the low-cost sensor minimum value at the NYSM Stony Brook site was notably higher than the NYSDEC Flax Pond reference site (Figure S16), potentially due to differences in their local environments - NYSM Stony Brook site is a commercial/suburban area 0.15 km ENE of NYS Route 25A while NYSDEC Flax Pond is a rural site on the south shore of Flax Pond (Figure S15). Alternatively, some of this difference may have been an artifact of applying an RF calibration model trained at an urban site with higher minimum concentrations to a suburban site with lower minimum concentrations. We note that the difference between the minimum concentrations at the NYSM Stony Brook and NYSDEC Flax Pond sites was roughly 1.6 ppb, much smaller than the fLOD (7.8, Table 4).

### 3.4 Comparison with Other Studies

The performance of the NCA-calibrated low-cost sensors was compared with two studies that were similar in scale and duration (Table 3); additional comparisons with more studies^5,40,41^ are included in Tables S7–S11. While the low-cost sensor calibration approaches for the 4 studies in Table 3 differed, they all focused on reducing the effort required for calibrations, thereby improving network scalability. The M19 approach defined a “typical” package as the median of a subset of packages that were simultaneously colocated with reference monitors. For each pollutant, a single general calibration model was then calculated from the typical package data and applied across the network. A key difference between the NCA and M19 is that the NCA accounts for the unique response of *each sensor in each package* by mapping its output signal to the corresponding variable in the keystone package using the SLR transfer functions (Figure 2), thus accounting for variations in the low-cost sensor response.

The Berkeley Environmental Air Quality and CO_2_ Network (BEACO_2_N), located in the San Francisco Bay Area, was comparable to our network in the number of sites; however, our network covered a larger geographical area and included rural sites. The calibration approach described in W25 did not require colocation of low-cost sensors with reference instruments; instead, MLR coefficients were trained monthly using data from a network of non-colocated reference instruments during periods when pollutant concentrations were determined to be similar at the reference sites. The W25 remote calibrations performed similarly to the NCA for NO_2_ and NO and slightly better for the corresponding values for O_3_. For CO, the NCA had a higher MAE (35.2 ppb) than the W25 calibrations (20.2 ppb), which may have reflected the higher ambient CO values in NYC (259.9 ppb) compared to San Francisco (158.0 ppb). We note that the W25 approach relied upon a network of reference instruments. This approach would be challenging in the NYCMA (Figure 1a), where many Hudson Valley sites located north of NYC are over 150 km from a reference site measuring all 5 target pollutants.

W25 raised questions about colocation calibration approaches that can be addressed using the NCA results, including sensor drift after deployment from the calibration site to field sites, and the transferability of calibrations developed at the calibration site to field sites. The ability of the NCA to account for seasonal and sensor drift was demonstrated in Section 3.2.3 using 6 months of hourly data from the evaluation package at the calibration site (Figure 6), and using data from colocation 2 that included 25 packages that were returned to the calibration site after field deployment (Table 2). The transferability of the NCMs derived from the keystone package to the field packages was facilitated by the SLRs that mapped each field package low-cost sensor to its counterpart in the keystone package (Figure S5) and demonstrated by the nearby field evaluations (Table S6). Further, unlike the W25 approach, the NCA relies upon *in situ* reference data for training the NCMs and therefore does not require assumptions regarding the spatial homogeneity of pollutant concentrations to train calibration models at field sites based on remotely located reference sites. We note that, even when sparsely located reference sites show agreement in pollutant concentrations, small-scale spatial variability, which is a common rationale for deploying dense low-cost sensor networks, may exist due to microclimates and local pollutant sources/sinks. Future studies could consider the implementation of a combined NCA and W25 approach that leverages each of their strengths.

### 3.5 Low-Cost Sensor EPA Monitoring Tiers

The EPA has provided performance metrics to define 4 “tiers” for low-cost sensors that correspond to their usefulness for various monitoring applications, with higher tier designations corresponding to better performance and greater utility.^42^ Calibrated low-cost sensors can be assigned tiers based on their precision and bias values. The precision was estimated using colocation 1 and 2 data by dividing the hourly standard deviation of simultaneously reporting sensors by their mean, with the average hourly precision reported in Table 4. Bias was estimated as the average NMBE of colocation 1 and 2 (Table 3). The NCA-calibrated PM_2.5_, O_3_, NO_2_, and CO meet the criteria for “Supplemental Monitoring” (Tier III), deemed of adequate quality to complement existing reference monitors and “fill in” monitoring gaps. NO met the criteria for “Hotspot Identification” (Tier II) and “Personal Exposure” (Tier IV). We note that the EPA does not provide guidance for low-cost NO sensor use in Supplemental Monitoring (Tier III); as such, all NCA-calibrated sensors in this study achieved the highest performance tier possible for low-cost sensors.

### 3.6 NCA Variations and Longer-Term Performance

In many cases, the calibration approach for low-cost air quality sensors will be dependent upon resources, such as the existence of (and access to) a reference site or multiple reference sites within the study region. The configuration of the calibration models also entails options with regard to predictor variables and training/testing/evaluation scenarios. For example, we could have used *all* of the keystone package data as training data (with no testing data), or used the *combined keystone and evaluation package* data as training data, as completed in an early iteration of the NCA detailed by Hojeily (2025).^39^ We computed the NCA for various scenarios, including a rigorous cross validation approach, and found relatively minor NCA performance sensitivity and impact on pollutant concentrations and evaluation statistics. Because the NCA was robust to these choices, we opted to present a straightforward and easily understood approach, splitting the keystone package data into training (75%) and testing (25%) portions and using the second long-term package as an evaluation data set.

The NCA showed good performance over the 16-month study period, accounting for seasonal sensor drift and electrochemical sensor degradation (Figure 6). While 16 months is longer than most reported low-cost sensor data sets in the literature, the performance beyond the manufacturer-specified 2-year lifetime of the electrochemical sensors remains an outstanding question. A recent study from the BEACO_2_N group found that the W25 method of updating calibrations monthly was effective at extending the useful lifetime of Alphasense sensors to at least 3 years.^43^ There remains a need for additional studies with longer data sets to evaluate how the low-cost sensors degrade, how to determine when they need to be replaced, and how effective calibration approaches, such as the NCA, are in accounting for diminished sensor performance. Additionally, more work is needed to quantify low-cost sensor calibration performance in different environments (i.e., urban, suburban, and rural).

## Supplementary Material



## Notes

Calibrated data included in this manuscript are version 1.3; Meteorological data from Queens, NY, and the calibrated data from the 38 field sites are accessible from the New York State Mesonet Website (www.nysmesonet.org); Data for the keystone package, the calibration model files, performance by package, and the transfer function coefficients are archived in an online repository (Zenodo DOI: 10.5281/zenodo.16989885, direct access via zenodo.org/records/16989885); For additional code and/or data inquiries, please contact ehojeily@albany.edu (PDF)

## References

[1] Bousiotis D., Allison G., Beddows D. C. S., Harrison R. M., Pope F. D. (2023). Towards
Comprehensive Air Quality Management Using Low-Cost Sensors for Pollution
Source Apportionment. Npj Clim. Atmospheric
Sci..

[2] Hodoli C. G., Mead I., Coulon F., Ivey C. E., Tawiah V. O., Raheja G., Nimo J., Hughes A., Haug A., Krause A., Amoah S., Sunu M., Nyante J. K., Tetteh E. N., Riffault V., Malings C. (2025). Urban Air
Quality Management
at Low Cost Using Micro Air Sensors: A Case Study from Accra, Ghana. ACS EST Air.

[3] Considine E. M., Braun D., Kamareddine L., Nethery R. C., deSouza P. (2023). Investigating
Use of Low-Cost Sensors to Increase Accuracy and Equity of Real-Time
Air Quality Information. Environ. Sci. Technol..

[4] Mead M. I., Popoola O. A. M., Stewart G. B., Landshoff P., Calleja M., Hayes M., Baldovi J. J., McLeod M. W., Hodgson T. F., Dicks J., Lewis A., Cohen J., Baron R., Saffell J. R., Jones R. L. (2013). The Use of Electrochemical
Sensors for Monitoring Urban Air Quality in Low-Cost, High-Density
Networks. Atmos. Environ..

[5] Levy
Zamora M., Buehler C., Lei H., Datta A., Xiong F., Gentner D. R., Koehler K. (2022). Evaluating the Performance
of Using Low-Cost Sensors to Calibrate for Cross-Sensitivities in
a Multipollutant Network. ACS EST Eng..

[6] Buehler, C. ; Xiong, F. ; Zamora, M. L. ; Skog, K. ; Kohrman-Glaser, J. ; Colton, S. ; McNamara, M. ; Ryan, K. ; Redlich, C. ; Bartos, M. ; Wong, B. ; Kerkez, B. ; Koehler, K. ; Gentner, D. Stationary and Portable Multipollutant Monitors for High Spatiotemporal Resolution Air Quality Studies Including Online Calibration Atmospheric Meas. Technol. Discuss. 2020, pp 1–28 10.5194/amt-2020-217. PMC9074123 35529304

[7] Zimmerman N., Presto A. A., Kumar S. P. N., Gu J., Hauryliuk A., Robinson E. S., Robinson A. L. (2018). R. Subramanian. A Machine Learning
Calibration Model Using Random Forests to Improve Sensor Performance
for Lower-Cost Air Quality Monitoring. Atmospheric
Meas. Technol..

[8] Castell N., Dauge F. R., Schneider P., Vogt M., Lerner U., Fishbain B., Broday D., Bartonova A. (2017). Can Commercial
Low-Cost Sensor Platforms Contribute to Air Quality Monitoring and
Exposure Estimates?. Environ. Int..

[9] Gonzalez, A. ; Boies, A. ; Swason, J. ; Kittelson, D. Field Calibration of Low-Cost Air Pollution Sensors Gases/In Situ Measurement/Instruments and Platforms; preprint 2019 10.5194/amt-2019-299.

[10] Malings C., Tanzer R., Hauryliuk A., Kumar S. P. N., Zimmerman N., Kara L. B., Presto A. A. (2019). R. Subramanian.
Development of a
General Calibration Model and Long-Term Performance Evaluation of
Low-Cost Sensors for Air Pollutant Gas Monitoring. Atmospheric Meas. Technol..

[11] Concas F., Mineraud J., Lagerspetz E., Varjonen S., Liu X., Puolamäki K., Nurmi P., Tarkoma S. (2021). Low-Cost Outdoor Air
Quality Monitoring and Sensor Calibration: A Survey and Critical Analysis. ACM Trans. Sens. Netw..

[12] Winter A. R., Zhu Y., Asimow N. G., Patel M. Y., Cohen R. C. (2025). A Scalable Calibration
Method for Enhanced Accuracy in Dense Air Quality Monitoring Networks. Environ. Sci. Technol..

[13] Clements, A. ; Duvall, R. ; Greene, D. ; Dye, T. Enhanced Air Sensor Guidebook. U.S. Environmental Protection Agency, Washington, DC, 2022 https://cfpub.epa.gov/si/si_public_record_report.cfm?Lab=CEMM&dirEntryId=356426.

[14] Snyder E. G., Watkins T. H., Solomon P. A., Thoma E. D., Williams R. W., Hagler G. S. W., Shelow D., Hindin D. A., Kilaru V. J., Preuss P. W. (2013). The Changing Paradigm
of Air Pollution Monitoring. Environ. Sci. Technol..

[15] Bagkis E., Hassani A., Schneider P., DeSouza P., Shetty S., Kassandros T., Salamalikis V., Castell N., Karatzas K., Ahlawat A., Khan J. (2025). Evolving Trends in Application of
Low-Cost Air Quality Sensor Networks: Challenges and Future Directions. npj Clim Atmos Sci..

[16] Duvall, R. M. ; Clements, A. L. ; Hagler, G. ; Kamal, A. ; Kilaru, V. ; Goodman, L. ; Frederick, S. ; Barkjohn, K. K. ; VonWald, I. ; Greene, D. ; Dye, T. Performance Testing Protocols, Metrics, and Target Values for Fine Particulate Matter Air Sensors: Use in Ambient, Outdoor, Fixed Site, Non-Regulatory Supplemental and Informational Monitoring Applications; EPA/600/R-20/280; United States Environmental Protection Agency, Office of Research and Development, Center for Environmental Measurement and Modeling: Research Triangle Park, NC 2021 https://cfpub.epa.gov/si/si_public_file_download.cfm?p_download_id=544837&Lab=CEMM.

[17] Duvall, R. M. ; Clements, A. L. ; Hagler, G. ; Kamal, A. ; Kilaru, V. ; Goodman, L. ; Frederick, S. ; Barkjohn, K. K. ; VonWald, I. ; Greene, D. ; Dye, T. Performance Testing Protocols, Metrics, and Target Values for Ozone Air Sensors: Use in Ambient, Outdoor, Fixed Site, Non-Regulatory Supplemental and Informational Monitoring Applications; EPA/600/R-20/279; United States Environmental Protection Agency, Office of Research and Development, Center for Environmental Measurement and Modeling: Research Triangle Park, NC 2021 https://cfpub.epa.gov/si/si_public_file_download.cfm?p_download_id=542101&Lab=CEMM.

[18] Barkjohn K. K., Gantt B., Clements A. L. (2021). Development
and Application of a
United States-Wide Correction for PM_2.5_ Data Collected
with the PurpleAir Sensor. Atmospheric Meas.
Technol..

[19] Brotzge J. A., Wang J., Thorncroft C. D., Joseph E., Bain N., Bassill N., Farruggio N., Freedman J. M., Hemker K., Johnston D., Kane E., McKim S., Miller S. D., Minder J. R., Naple P., Perez S., Schwab J. J., Schwab M. J., Sicker J. (2020). A Technical Overview of the New York
State Mesonet Standard Network. J. Atmospheric
Ocean. Technol..

[20] Miller S. D., Hojeily E.H., Covert J.M., Lu C.-H., Bari M. A., Schwab M. J., Moore C., Brooking M. (2025). Combining Low-Cost
Sensors with the New York State Mesonet for Continuous Fine-Scale
Air Quality Monitoring in New York City. Environ.
Sci. Technol. - Air.

[21] Ambient Air Monitoring Network 5 Year Assessment 2025; New York State Department of Environmental Conservation: Albany, NY, 2025 pp. 1-77. https://dec.ny.gov/sites/default/files/2025-05/2025fiveyearassessment.pdf (accessed Sept 10, 2025).

[22] Hayward I., Martin N. A., Ferracci V., Kazemimanesh M., Kumar P. (2024). Low-Cost Air Quality Sensors: Biases,
Corrections and Challenges
in Their Comparability. Atmosphere.

[23] Wang A., Machida Y., deSouza P., Mora S., Duhl T., Hudda N., Durant J. L., Duarte F., Ratti C. (2023). Leveraging
Machine Learning Algorithms to Advance Low-Cost Air Sensor Calibration
in Stationary and Mobile Settings. Atmos. Environ..

[24] Civerolo K. L., Rattigan O. V., Felton H. D., Schwab J. J. (2017). Changes in Gas-Phase
Air Pollutants across New York State, USA. Aerosol
Air Qual. Res..

[25] Li J., Hauryliuk A., Malings C., Eilenberg S. R., Subramanian R., Presto A. A. (2021). Characterizing the Aging of Alphasense
NO _2_ Sensors in Long-Term Field Deployments. ACS Sens..

[26] Ouimette J., Arnott W. P., Laven P., Whitwell R., Radhakrishnan N., Dhaniyala S., Sandink M., Tryner J., Volckens J. (2024). Fundamentals
of Low-Cost Aerosol Sensor Design and Operation. Aerosol Sci. Technol..

[27] Holder A. L., Mebust A. K., Maghran L. A., McGown M. R., Stewart K. E., Vallano D. M., Elleman R. A., Baker K. R. (2020). Field Evaluation
of Low-Cost Particulate Matter Sensors for Measuring Wildfire Smoke. Sensors.

[28] Laurent J. G. C., Parhizkar H., Calderon L., Lizonova D., Tsiodra I., Mihalopoulos N., Kavouras I., Alam M., Baalousha M., Bazina L., Kelesidis G. A., Demokritou P. (2024). Physicochemical
Characterization of the Particulate Matter in New Jersey/New York
City Area, Resulting from the Canadian Quebec Wildfires in June 2023. Environ. Sci. Technol..

[29] Sayahi T., Butterfield A., Kelly K. E. (2019). Long-Term Field Evaluation of the
Plantower PMS Low-Cost Particulate Matter Sensors. Environ. Pollut..

[30] Andreae M. O. (2019). Emission
of Trace Gases and Aerosols from Biomass Burning – an Updated
Assessment. Atmos. Chem. Phys..

[31] Lee H., Jaffe D. A. (2024). Wildfire
Impacts on O3 in the Continental United States
Using PM2.5 and a Generalized Additive Model (2018–2023). Environ. Sci. Technol..

[32] Cowell N., Chapman L., Bloss W., Pope F. (2022). Field Calibration and
Evaluation of an Internet-of-Things-Based Particulate Matter Sensor. Front. Environ. Sci..

[33] Raheja G., Nimo J., Appoh E. K.-E., Essien B., Sunu M., Nyante J., Amegah M., Quansah R., Arku R. E., Penn S. L., Giordano M. R., Zheng Z., Jack D., Chillrud S., Amegah K., Subramanian R., Pinder R., Appah-Sampong E., Tetteh E. N., Borketey M. A., Hughes A. F., Westervelt D. M. (2023). Low-Cost Sensor Performance Intercomparison,
Correction Factor Development, and 2+ Years of Ambient PM_2.5_ Monitoring in Accra, Ghana. Environ. Sci.
Technol..

[34] Levy
Zamora M., Buehler C., Datta A., Gentner D. R., Koehler K. (2023). Identifying Optimal Co-Location Calibration Periods
for Low-Cost Sensors. Atmospheric Meas. Technol..

[35] Lu J., Liu A., Dong F., Gu F., Gama J., Zhang G. (2018). Learning under
Concept Drift: A Review. IEEE Trans. Knowl.
Data Eng..

[36] D’Elia G., Ferro M., Sommella P., Ferlito S., De Vito S., Di Francia G. (2024). Concept Drift
Mitigation in Low-Cost Air Quality Monitoring
Networks. Sensors.

[37] D’Elia G., Ferro M., Sommella P., De Vito S., Ferlito S., D’Auria P., Francia G. D. (2022). Influence of Concept Drift on Metrological
Performance of Low-Cost NO_2_ Sensors. IEEE Trans. Instrum. Meas..

[38] Alphasense . NO2-B43F/NO2-B43F+ Nitrogen Dioxide Sensor 2024 https://ametekcdn.azureedge.net/mediafiles/project/oneweb/oneweb/alphasense/products/datasheets/alphasense_no2-b43f_datasheet_en_4.pdf.

[39] Hojeily, E. Calibration of a Low-Cost Air Quality Sensor Package Integrated into the New York State Mesonet. Masters Thesis, University at Albany, State University of New York: Albany, NY, 2025 https://scholarsarchive.library.albany.edu/etd/153.

[40] Cross E. S., Williams L. R., Lewis D. K., Magoon G. R., Onasch T. B., Kaminsky M. L., Worsnop D. R., Jayne J. T. (2017). Use of Electrochemical
Sensors for Measurement of Air Pollution: Correcting Interference
Response and Validating Measurements. Atmospheric
Meas. Technol..

[41] deSouza P., Kahn R., Stockman T., Obermann W., Crawford B., Wang A., Crooks J., Li J., Kinney P. (2022). Calibrating
Networks of Low-Cost Air Quality Sensors. Atmospheric
Meas. Technol..

[42] Williams, R. ; Kilaru, V. ; Snyder, E. ; Kaufman, A. ; Dye, T. ; Rutter, A. ; Russell, A. ; Hafner, H. Air Sensor Guidebook, EPA/600/R-14/159; U.S. Environmental Protection Agency, 2014. https://nepis.epa.gov/Exe/ZyPURL.cgi?Dockey=P100JDZI.txt

[43] Winter A. R., Zhu Y., Asimow N. G., Patel M. Y., Cohen R. C. (2025). Sustained Performance
of Low-Cost Air Quality Sensors in Long-Term Deployments. ACS Sens..

[44] QGIS Development Team . QGIS Geographic Information System, Ver. 3.32.2-Lima; QGIS Association, http://www.qgis.org (accessed Sept 24, 2025).

[45] World_Imagery (MapServer) [QGIS Basemap] . ArcGIS REST Services Directory; Esri, https://server.arcgisonline.com/arcgis/rest/services/World_Imagery/MapServer (accessed Sept 24, 2025).

[46] U.S. States and Territories [Shapefile] . NWS GIS Portal. National Weather Service, National Oceanic and Atmospheric Administration, U.S. Dept of Commerce, https://www.weather.gov/gis/USStates (accessed Sept 24, 2025).

