# Correction to “A Network Calibration Approach Improves the Accuracy and Long-Term Stability of a Low-Cost Air Quality Mesonet in New York City”

**DOI:** 10.1021/acsestair.6c00297

**Published:** 2026-09-28

**Authors:** Ellie H. Hojeily, Jason M. Covert, Margaret J. Schwab, Clover Moore, Cheng-Hsuan Lu, Md. Aynul Bari, Scott D. Miller

In the original manuscript, we stated that the coefficient of determination (*R* ^2^) was the metric used to describe model performance in sections 2.3.3 and 3.2.1; however, the actual metric used was the Pearson *r*-squared (*r* ^2^). The Pearson *r*-squared measures the strength of linear correlation and ignores biases, while the coefficient of determination quantifies how variance in observations is explained by the model and penalizes biases. This error does not change the conclusions of the original manuscript. In discovering this error, we realized that some previous studies reported *R* ^2^ while others reported *r* ^2^. To address this and allow fair comparisons with similar studies, a corrected version of Table 3 appears below.

Corrected Tables S7−S11 corresponding to section S7 of the original Supporting Information have been provided. As with Table 3, all tables now include both *R* ^2^ and *r* ^2^ values for our study and proper identification of *R* ^2^ and *r* ^2^ values reported in the studies used for comparison.

**Table 3:** Comparison with Similar Work for the NCA Validated at the NYSDEC Queens College Calibration Site[Table-fn t1fn88]

pollutant	study	mean reference concentration	*R* ^2^	*r* ^2^	MAE	MBE
PM_2.5_	this study	9.3 ± 0.3 μg m^−3^	0.88 ± 0.02	0.90 ± 0.01	1.3 ± 0.1 μg m^−3^	−0.0 ± 0.2 μg m^−3^
	Barkjohn et al., 2021^d^	4−10 μg m^−3^	0.82	NR^a^	1.8 μg m^−3^	0 μg m^−3^
	Raheja et al., 2023^e^	5.2−60 μg m^−3^	0.89	NR^a^	1.9 μg m^−3^	NR^a^
O_3_	this study	33.5 ± 1.2 ppb	0.79 ± 0.02	0.84 ± 0.02	4.9 ± 0.3 ppb	−1.1 ± 0.5 ppb
	W25^b^	26.8 ppb	0.95 (0.88)	NR^a^	1.9 (2.9) ppb	−0.3 (0.0) ppb
	M19^a^	4−9 ppb	NR^a^	0.73 ± 0.12	5.9 ± 1.5 ppb	0.9 ± 2.9 ppb
NO_2_	this study	11.1 ± 0.5 ppb	0.77 ± 0.04	0.82 ± 0.02	2.9 ± 0.2 ppb	0.1 ± 0.3 ppb
	W25^b^	6.4 ppb	0.82 (0.66)	NR^a^	1.7 (2.4) ppb	0.3 (0.9) ppb
	M19^a^	4−9 ppb	NR^a^	0.30 ± 0.17	3.4 ± 0.5 ppb	0.2 ± 2.6 ppb
NO	this study	2.4 ± 0.4 ppb	0.74 ± 0.05	0.81 ± 0.04	1.2 ± 0.1 ppb	0.4 ± 0.2 ppb
	W25^b^	2.4 ppb	0.82 (0.79)	NR^a^	1.0 (1.1) ppb	0.3 (0.4) ppb
	M19^a^	1−3 ppb	NR^a^	0.17 ± 0.08	13.2 ± 22.7 ppb	9.9 ± 20.1 ppb
CO	this study	259.9 ± 6.7 ppb	0.81 ± 0.03	0.87 ± 0.03	35.2 ± 1.9 ppb	9.4 ± 5.7 ppb
	W25^b^	158.0 ppb	0.90	NR^a^	20.2 ppb	−1.5 ppb
	M19^a^	145−451 ppb	NR^a^	0.85 ± 0.09	56.0 ± 8.0 ppb	6.0 ± 93 ppb

a More comprehensive tables for each pollutant can be found in Tables S7−S11.

b Statistics taken from Malings et al. (2019) (Table S4) for their general hybrid “HY” model and reference range from their Table 4. Results are presented as the average ± standard deviation. They present the Pearson *r*-squared in their work.

c Statistics taken from Winter et al. (2025) for their colocated (remote) calibration models, except for CO where only the colocated model is reported.

d Statistics taken from Barkjohn et al. (2021) (Table S8) for their “2+RH” correction model evaluated using a leave out by date validation.

e Statistics taken from Raheja et al. (2023) (Table 3) from their PurpleAir RF model. Additional statistics for their other models (i.e., MLR and XGBoost) are provided in Table S9.

f Not reported.

## Supplementary Material



## Notes

Tables S7−S11 (PDF)

