# Supplementary material for: A Network Calibration Approach Improves the Accuracy and Long-Term Stability of a Low-Cost Air Quality Mesonet in New York City
Source: ACS EST Air. 2025 Dec 25;3(1):58–72. doi: 10.1021/acsestair.5c00205 (PMC12797186; doi:10.1021/acsestair.5c00205)
Supplement: Supplementary file 1 [file ea5c00205_si_001.pdf]

Supplemental Information

A Network Calibration Approach Improves the Accuracy and Long-Term Stability of a Low-Cost Air Quality Mesonet in New York City

Ellie H. Hojeily[a]*, Jason M. Covert[a], Margaret J. Schwab[a], Clover Moore[a], Cheng-Hsuan Lu[a,b], Md. Aynul Bari[c] , Scott D. Miller[a]

[a]*Atmospheric Sciences Research Center, University at Albany, Albany, New York, 12226, United States*
[b]*Joint Center for Satellite Data Assimilation, University Corporation for Atmospheric Research, Boulder, Colorado, 80301, United States*
[c]*Department of Environmental and Sustainable Engineering, University at Albany, Albany, New York, 12226, United States*

* Email: ehojeily@albany.edu

This file includes:

Supplementary Figures S1 to S16

Supplementary Tables S1 to S11

This document is organized by section:

- Section S1: Sensor package and calibration site overview
- Section S2: Calibration model development
- Section S3: SLR transfer function overview
- Section S4: Package deployments to calibration site
- Section S5: Calibration performance evaluation across environmental conditions
- Section S6: Calibration performance following relocation to select NYSM sites
- Section S7: Comparison with similar studies

Number of Pages: 28

Section S1: Sensor package and calibration site overview

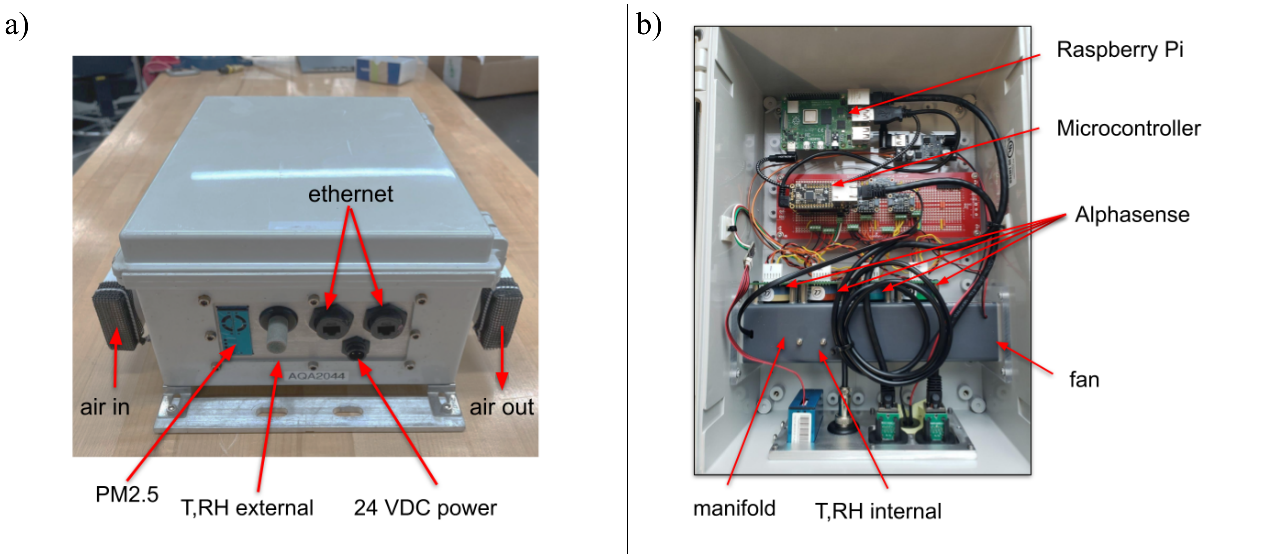

Figure S1. UAlbany air quality sensor package: a) external view; and b) internal view showing the gray PVC sensor manifold where Alphasense electrochemical sensors were installed. Additional details are provided in Miller et al. (in press).

Table S1. Low-cost sensor manufacturer and model numbers, NYSDEC Queens College calibration site reference instruments, and the median and range of reference sensor pollutant concentrations during the 16-month period from 28 April 2023 to 31 Aug 31 2024.

	**$PM_{2.5}$**	**O_3**	**Ox (O_3NO_2)**	**NO_2**	**NO**	**CO**
**Low-cost sensor**	Plantower PMS5003	Alphasense OX-B431, NO2-B43F	Alphasense OX-B431	Alphasense NO2-B43F	Alphasense NO-B4	Alphasense CO-B4
**Reference Instrument**	Teledyne API T640	Teledyne API T400 Ozone Analyzer	N/A	Thermo Electron 42C-TL, 42i-TL	Teledyne API 200 EU/501	Thermo Electron 48i-TLE
**EPA AQS Parameter Identifier**	88101 ($PM_{2.5}$ Local Conditions)	44201	N/A	42602	42603	42101
**Median reference concentration**	13.65 μgm^{-3} 5.92* μgm^{-3}	29.0 ppb	39.9 ppb	8.5 ppb	0.70 ppb	231.ppb
**Range of reference concentrations**	0.3 - 411.1 (45.6*) μgm^{-3}	0.0 - 99.0 ppb	11.5 - 105.7 ppb	0.0 - 64.6 ppb	0.0 - 322.2 ppb	62.0 - 1952.0 ppb

* 2023 Canadian Wildfire data removed

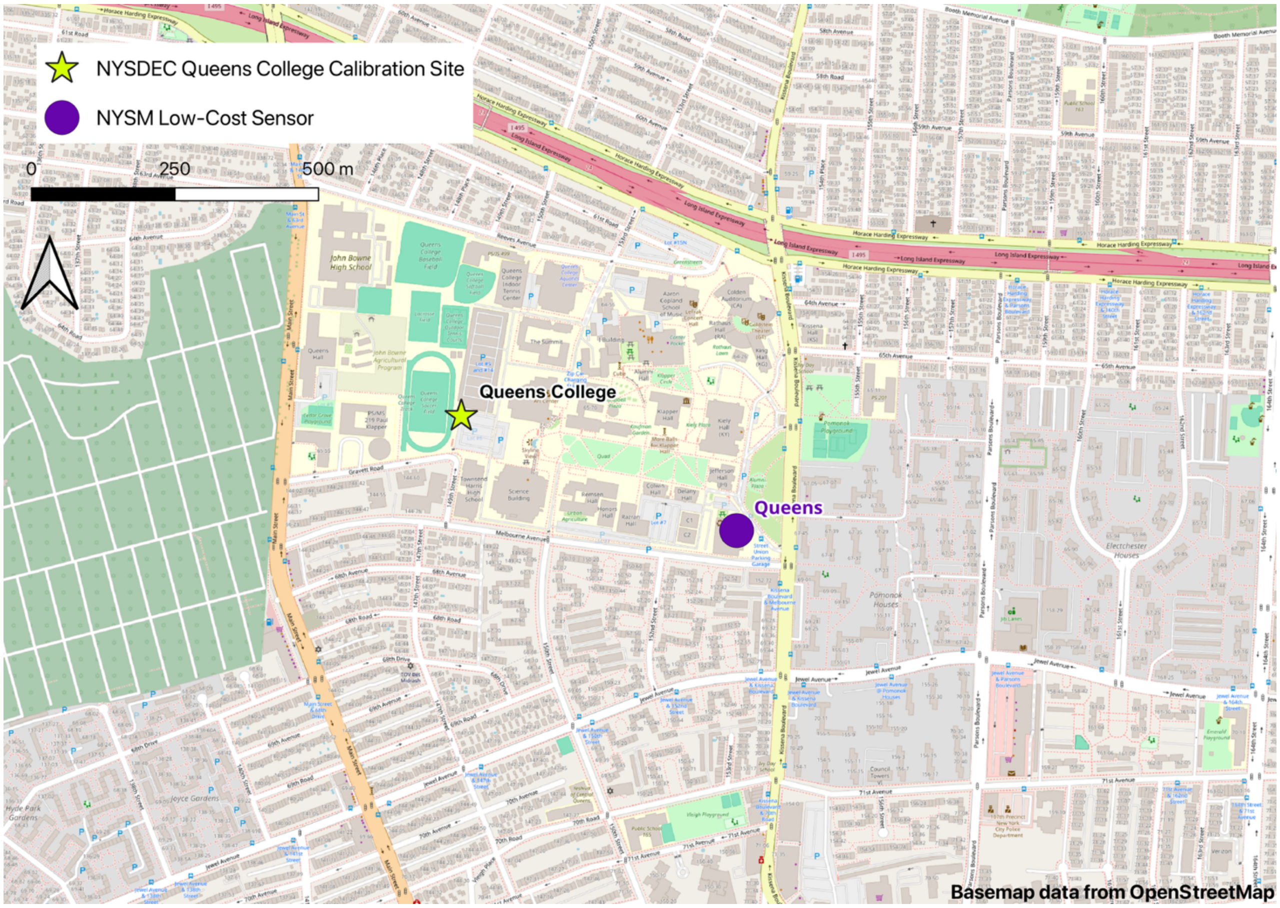

Figure S2. Map depicting the NYSDEC Queens College calibration site (yellow star) and the NYSM Queens site (purple dot). Both sites are located on the Queens College campus in Queens, NY. The distance between the sites is approximately 0.57 km. The basemap was sourced from OpenStreetMap and the map was created in QGIS.

Section S2: Calibration model development

Below are the MLR calibration coefficient and offset values for the Network Calibration Models (NCM). We note that the SLR transfer functions should be applied *prior* to application of the NCMs, especially for the electrochemical sensors (see Section S3).

***$PM_{2.5}$*:**

$$\mathrm{PM}_{2.5} = 6.37 + 0.42 \cdot \mathrm{PM}_{2.5\mathrm{raw}} - 0.05 \cdot \mathrm{RH}$$

***O_3*:**

$$\mathrm{O}_{3_{\mathrm{ref}}} = 2.34 + 0.91 \cdot \mathrm{O}_{3_{\mathrm{EST}}} + 0.09 \cdot \mathrm{T} - 0.02 \cdot \mathrm{RH}$$

***NO_2*:**

$$\mathrm{NO}_{2\mathrm{ref}} = -19.5 + 2113.29 \cdot \mathrm{NO}_{2\mathrm{raw}} - 0.08 \cdot \mathrm{RH} + 0.24 \cdot \mathrm{T} - 649.18 \cdot \mathrm{O}_3\mathrm{NO}_{2\mathrm{raw}}$$

***NO*:**

$$\mathrm{NO}_{\mathrm{ref}} = 7.73 + 2198.43 \cdot \mathrm{NO}_{\mathrm{raw}} - 0.35 \cdot \mathrm{T} + 51.41 \cdot \mathrm{T} \cdot \mathrm{NO}_{\mathrm{raw}} + 0.14 \cdot \mathrm{NO}_{2\mathrm{hybrid}} + 0.004 \cdot \mathrm{RH}$$

***CO*:**

$$\mathrm{CO}_{\mathrm{ref}} = 47.62 + 3432.09 \cdot \mathrm{CO}_{\mathrm{raw}}^2 + 1294.10 \cdot \mathrm{CO}_{\mathrm{raw}}$$

***O_3NO_2*:**

$$\mathrm{O3}_2\mathrm{NO}_{2\mathrm{ref}} = 36.22 + 1189.07 \cdot O_3\mathrm{NO}_{2raw} + 0.20 \cdot \mathrm{T} - 0.17 \cdot \mathrm{RH}$$

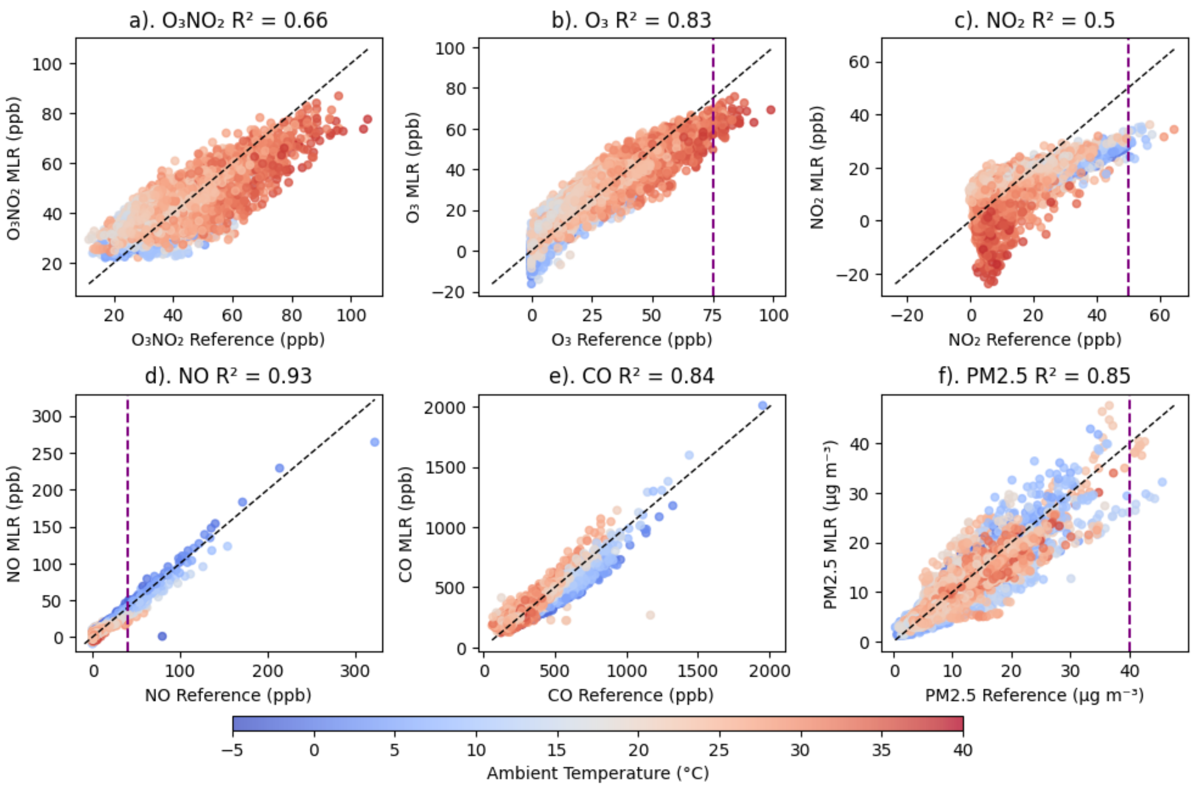

Figure S3. One year of MLR calibrated low-cost sensor observations from AQA2020 (y-axis) plotted against measurements from reference instruments (x-axis). The points are shaded by ambient temperature from -5° (blue) to 40°C (red). The RF thresholds (CTH) are plotted as a vertical purple dashed line O_3 (a), NO_2 (b), NO (d), and $PM_{2.5}$ (f).

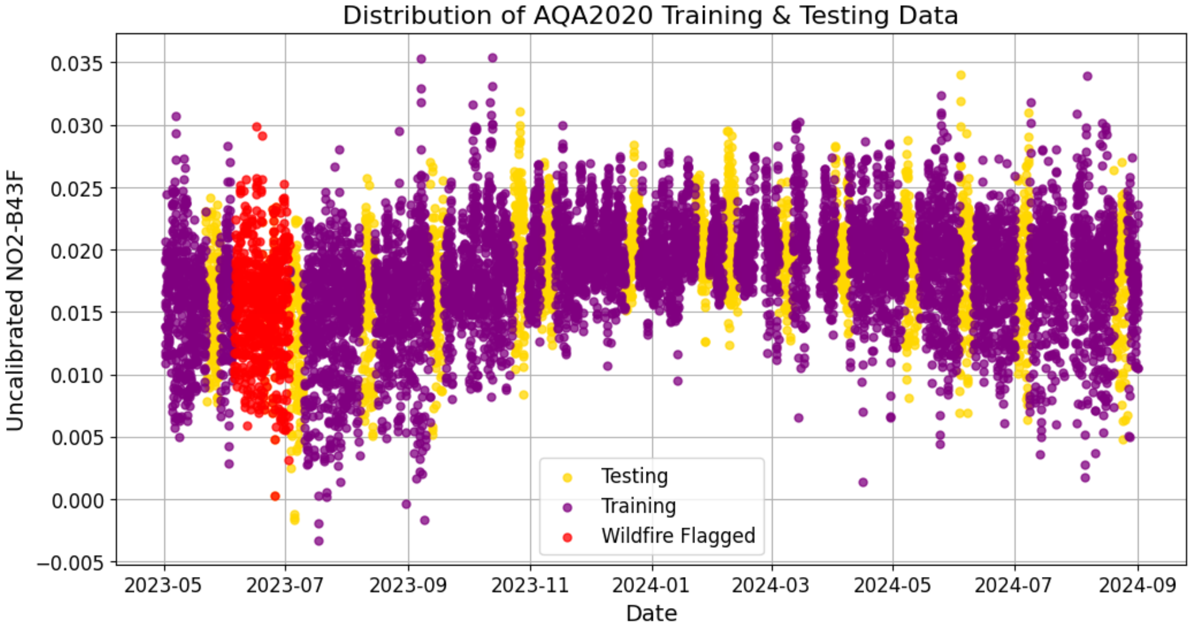

Figure S4. Distribution of training (purple) and testing (gold) data from the NCM keystone package, AQA2020. Each dot represents 1 hour of data. 1 week per month was randomly selected for testing. The red dots indicate the data removed due to extensive wildfire exposure. Identical training and testing dates were chosen for the rest of the pollutants.

Table S2. Network model performance on long-term calibration site packages AQA2020 (keystone) and AQA2053 (evaluation).The performance statistics are described in Section 2.3.4. The units for RMSE, MAE, and MBE are indicated in parenthesis under the pollutant name while $R^{2,}$ NMAE, and NMBE are unitless.

Pollutant	Package	R^2	RMSE	MAE	NMAE	MBE	NMBE
**$PM_{2.5}$ (µg m^{-3})**	AQA2020 (train)	0.96	1.2	0.7	0.08	-0.0	-0.00
	AQA2020 (test)	0.90	2.0	1.2	0.15	0.2	0.02
	AQA2053	0.90	1.8	1.2	0.13	-0.3	-0.04
**O_3 (ppb)**	AQA2020 (train)	0.96	3.1	2.3	0.08	-0.1	-0.00
	AQA2020 (test)	0.89	5.1	4.0	0.15	0.2	0.01
	AQA2053	0.87	6.3	4.5	0.13	-2.5	-0.07
**NO_2 (ppb)**	AQA2020 (train)	0.98	1.5	1.0	0.09	-0.1	-0.00
	AQA2020 (test)	0.93	2.8	2.1	0.16	0.0	0.00
	AQA2053	0.78	5.6	3.7	0.36	2.2	0.22
**O_3NO_2 (ppb)**	AQA2020 (train)	0.67	6.8	5.3	0.13	-0.0	0.00
	AQA2020 (test)	0.62	6.9	5.4	0.14	0.5	0.01
	AQA2053	0.74	6.3	4.8	0.11	-0.8	-0.02
**NO (ppb)**	AQA2020 (train)	0.98	1.6	0.8	0.14	-0.1	-0.03
	AQA2020 (test)	0.98	1.5	0.5	0.19	-0.2	-0.04
	AQA2053	0.93	1.9	1.0	0.44	0.5	0.25
**CO (ppb)**	AQA2020 (train)	0.84	48.3	34.9	0.13	-0.0	-0.00
	AQA2020 (test)	0.83	55.2	40.9	0.15	-8.4	-0.03
	AQA2053	0.85	39.5	27.3	0.11	5.7	0.02

Section S3: Simple Linear Regression (SLR) transfer functions

The SLR transfer functions map raw field package variables to the keystone package raw variables and are meant to account for potential voltage offsets or differences in sensitivity (gain) of the OEM sensors (Figure S6). Generally, package AQA2020 was used as the keystone package, except for 13 packages deployed prior to AQA2020 for which AQA2013 was used. Table S3 summarizes the R^2 and slope values calculated between the field packages and the keystone package prior to applying the SLR transfer functions. For PM2.5, the SLR transfer function slope values average to 1, indicating that the $PM_{2.5}$ sensor could likely have a NCM applied without need for a SLR transfer function. For the electrochemical sensors, the SLR average R^2 values ranged from 0.84-0.92 and their slopes ranged from 0.86-0.98.

The stability of the SLR transfer functions between co-locations 1 ("C1") and 2 ("C2") is tabulated in Table S5 by comparing the performance of the NCM during C2 using the SLR transfer function coefficient and offset values derived from C1 and C2, respectively. For all pollutants, the change in R2 between C1 and C2 was less than 0.04 and the changes in MAE between C1 and C2 were small (0.1 ug/m3 for PM2.5, 1.7 ppb for O3, 1.2 ppb for NO2, 0.6 ppb for NO, and 5.6 ppb for CO). The consistency between MAE in C1 and C2 signifies the SLRs were largely stable for the duration of their deployments.

Table S3. Performance statistics for the raw sensors prior to mapping to the keystone package. Data from 82 co-locations is tabulated and presented as the average +- the 95% confidence interval. Applying the SLR reduces slope and gain to 1.0 ± 0.0 and 0.0 ± 0.0, respectively.

Pollutant	R^2	Slope
**$PM_{2.5}$**	0.99 ± 0.00	1.0 ± 0.01
**O_3**	0.87 ± 0.02	0.86 ± 0.03
**NO_2**	0.87 ± 0.03	0.98 ± 0.04
**NO**	0.84 ± 0.04	0.97 ± 0.05
**CO**	0.92 ± 0.03	0.87 ± 0.03
**O_3NO_2**	0.88 ± 0.03	0.93 ± 0.03

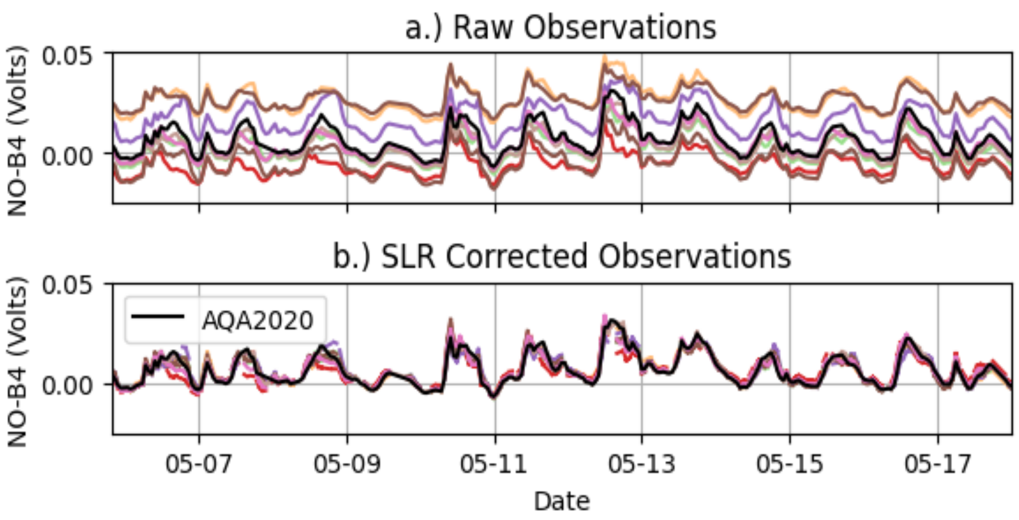

Figure S5. An example of the change in sensor output before (a) and after (b) mapping the field packages to the keystone package (AQA2020, black). Note the unique zero offsets among the simultaneous reporting packages in (a).

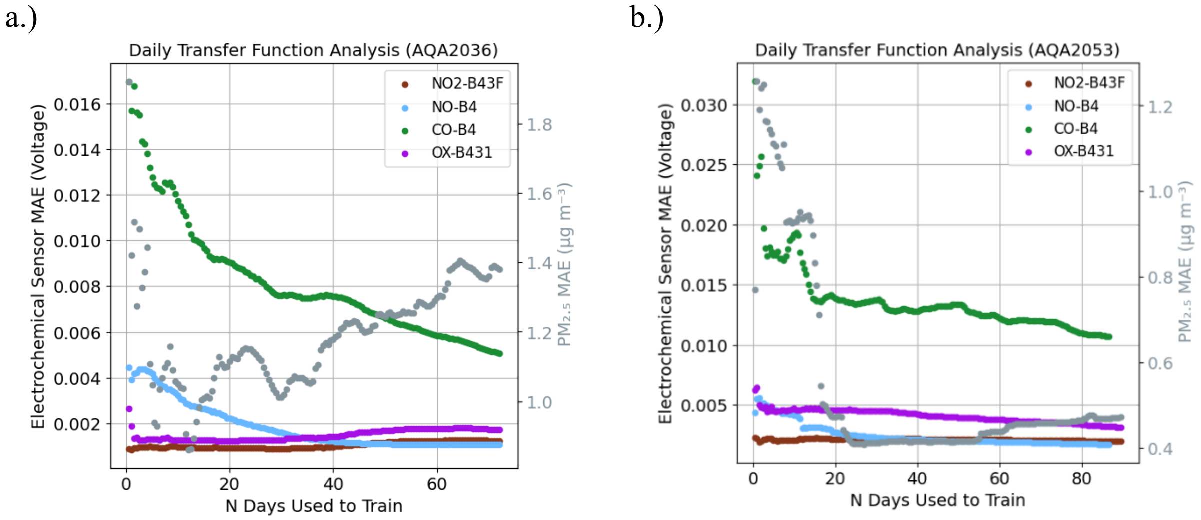

Figure S6. MAE as a function of days used to train the SLRs transfer functions for two field packages: a) AQA2036; and b) AQA2053. The first half of co-location data for the packages were used to train the models, with the latter half used to test. The training period iteratively increased by one day while the testing data was static. The number of co-location days were 194 for AQA2036 and 157 for AQA2053. These packages were chosen for this analysis due to having the longest co-location periods with the keystone package. Results for the electrochemical sensors are plotted on the left y-axis while the Plantower PMS5003 is plotted on the right y-axis in grey.

Table S4. Stability of the SLR transfer functions that mapped raw field package variables to the keystone package raw variables, assessed by comparing sensitivity of pollutant concentrations at the NYSDEC Queens College site during co-location 2 using SLRs determined for 25 packages before (co-location 1, C1) and after (co-location 2, C2) field site deployments.

**Pollutant**	**R^2 - C1**	**R^2 - C2**	**MAE - C1**	**MAE - C2**	**MBE - C1**	**MBE - C2**
**$PM_{2.5}$ (µg m^{-3})**	0.91 ± 0.02	0.91 ± 0.02	1.3 ± 0.1	1.2 ± 0.1	-0.1 ± 0.3	-0.3 ± 0.3
**O_3 (ppb)**	0.81 ± 0.04	0.85 ± 0.03	6.0 ± 1.1	4.3 ± 0.5	1.1 ± 2.0	-1.3 ± 0.9
**NO_2 (ppb)**	0.81 ± 0.04	0.82 ± 0.05	3.7 ± 0.5	2.9 ± 0.4	-0.4 ± 1.3	1.2 ± 0.6
**O_3NO_2 (ppb)**	0.60 ± 0.05	0.61 ± 0.05	5.6 ± 0.6	5.4 ± 0.5	0.6 ± 1.1	-0.5 ± 1.0
**NO (ppb)**	0.75 ± 0.11	0.77 ± 0.11	1.7 ± 0.5	1.1 ± 0.2	0.3 ± 0.7	-0.3 ± 0.2
**CO**	0.80 ± 0.08	0.80 ± 0.08	43.8 ± 6.7	38.2 ± 2.9	-15.7 ± 15.0	-5.7 ± 12.0

Section S4: Package deployments to calibration site

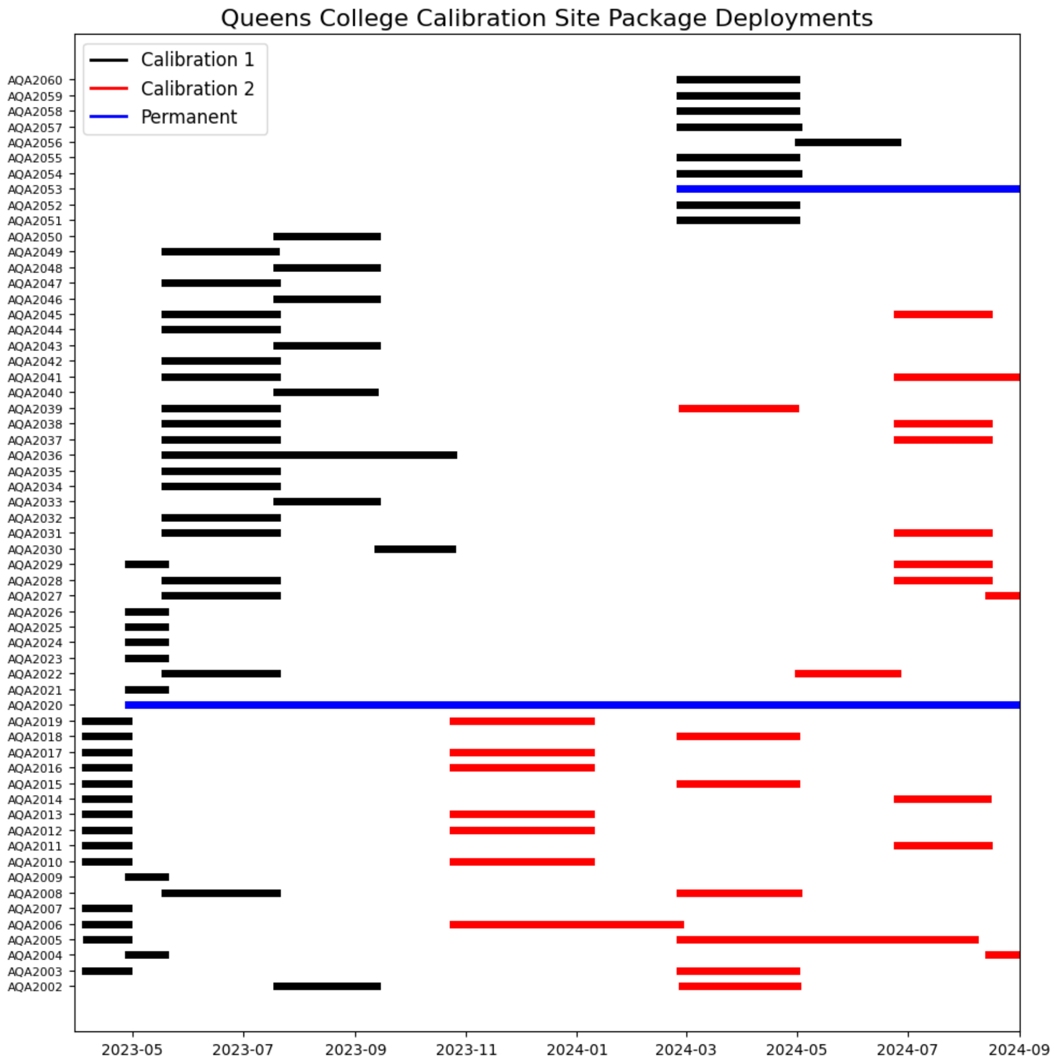

Figure S7. Overview of package deployments to the Queens College calibration site. Packages are plotted using their package identifiers (AQA20) on the y-axis. Each bar represents the amount of time spent by a package at the calibration site. Each bar is shaded by the calibration number; co-location 1 is shaded in black, co-location 2 is shaded in red, and packages permanently deployed at the calibration site are shaded in blue. For example, AQA2003 was first calibrated in May 2023 then recalibrated in March 2024. The keystone package (AQA2020) and long-term evaluation package (AQA2053) are plotted in blue.

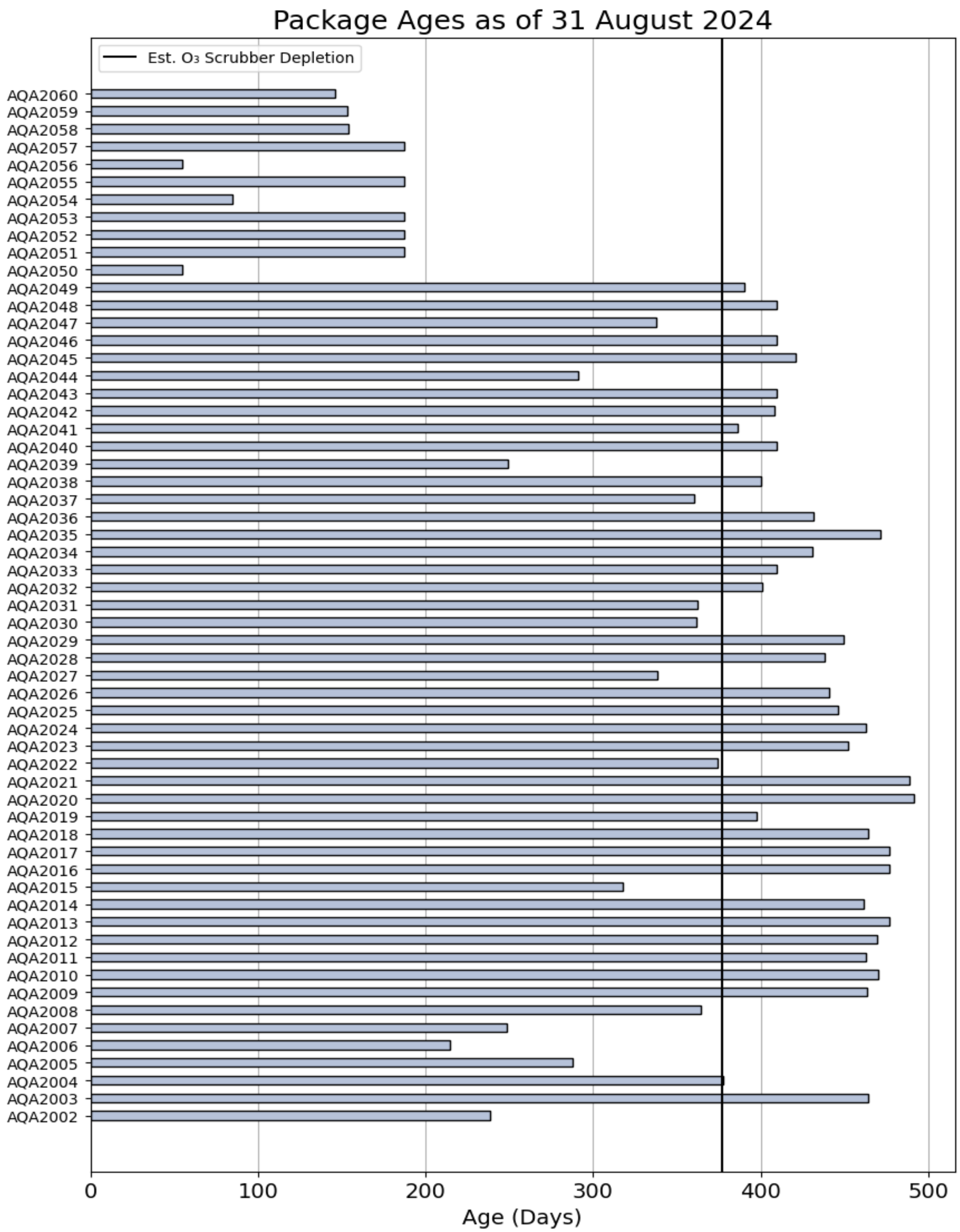

Figure S8. Age (days since deployment start) for the sensor packages. The estimated O_3 scrubber lifetime for the NO2-B43F calculated based the average O_3 concentration at Queens College is plotted as a vertical black line.

<u>Section S5: Calibration performance evaluation across environmental conditions</u>

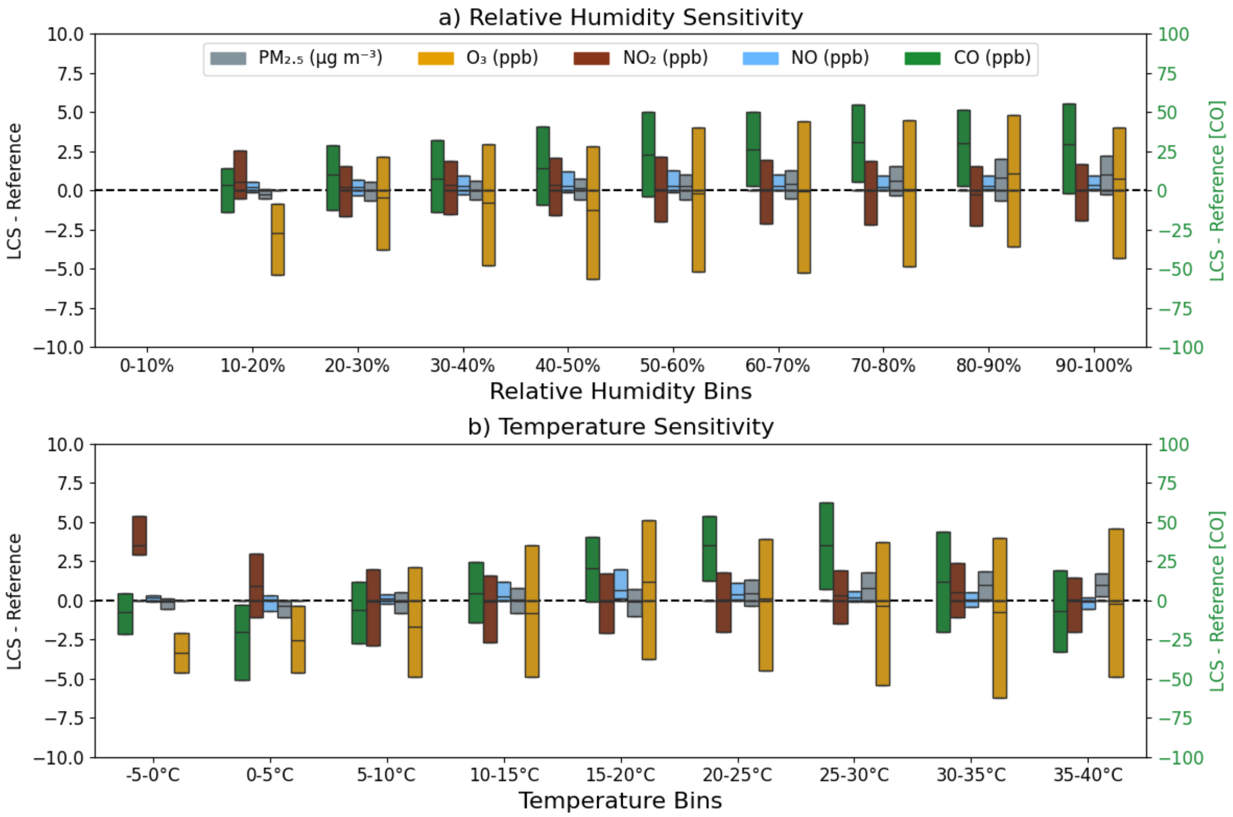

Figure S9. The distribution of error (low-cost sensor minus reference), aggregated by relative humidity (a) and temperature (b) bins. For each pollutant (various colors), the quartiles of bias per meteorological bin is plotted. CO is plotted on the right y-axis (green). Data from co-location 1, with the wildfire data omitted from $PM_{2.5}$, is plotted to isolate the impacts of aging. The boxplots were created using the seaborn boxplot function, with the boxes representing interquartile range of the dataset and the solid dashed line representing the median.

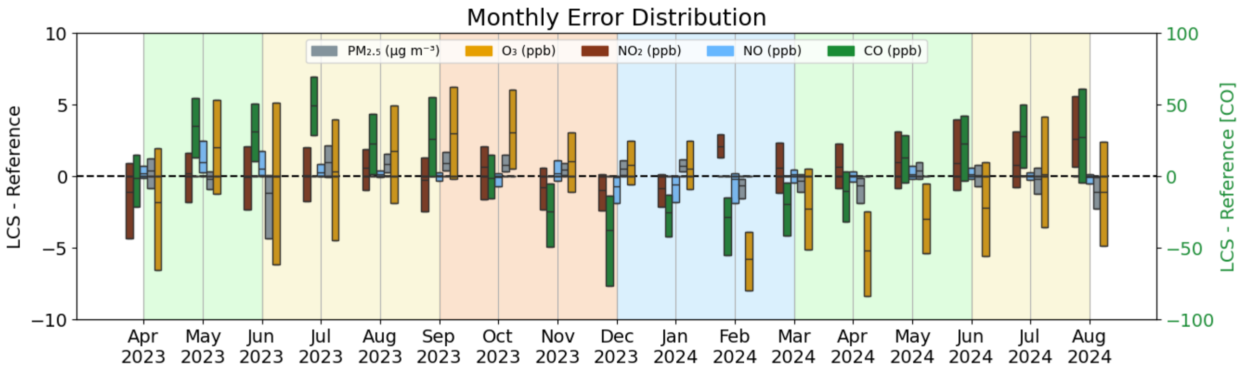

Figure S10. The distribution of error across aggregated by month across the 82 co-location periods collected during this study. Per month, the order of the bars is as follows: CO (green, ppb), NO_2 (maroon, ppb), NO (blue, ppb), PM2.5 (gray, µg m^{-3}), and O_3 (yellow, ppb). CO is plotted on the secondary y-axis. The shaded regions correspond to meteorological seasons: spring (green), summer (yellow), fall (orange), and winter (blue). The boxplots were created using the seaborn boxplot function, with the boxes representing interquartile range of the dataset and the solid dashed line representing the median.

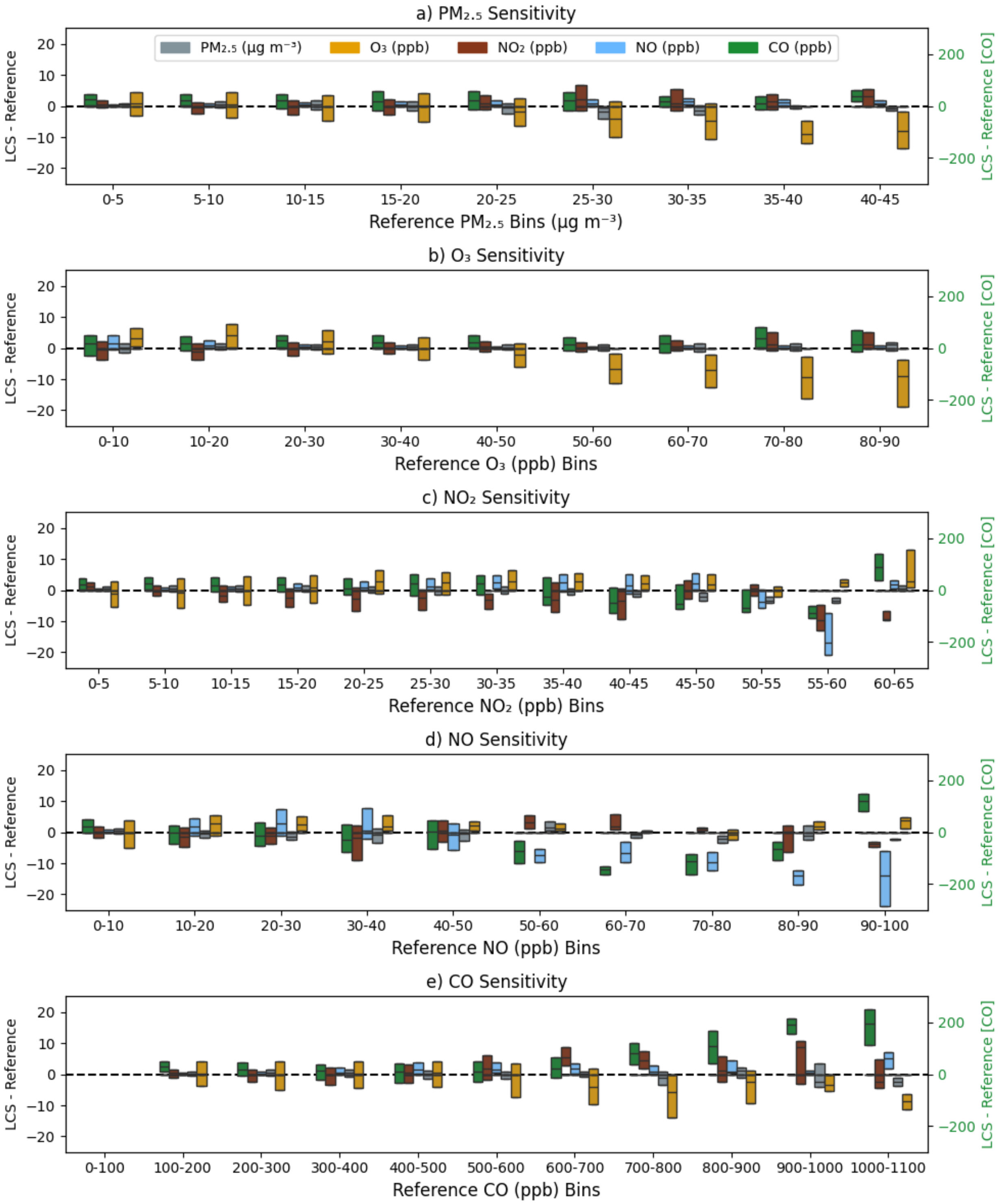

Figure S11. Same as Figure S10 except error is aggregated by reference observations at the NYSDEC Queens College site. The boxplots were created using the seaborn boxplot function, with the boxes representing interquartile range of the dataset and the solid dashed line representing the median.

Section S6: Calibration performance following relocation to select NYSM field sites

Table S5. NYSDEC Comparison Site Information. The EPA AQS site IDs were used to retrieve reference data from the EPA AQS API. More information about the API can be found at https://aqs.epa.gov/aqsweb/documents/data_api.html

Site Name	EPA AQS Site ID	Latitude	Longitude	Elevation (meters above mean sea level)
Queens College	36-081-0124	40.736140	-73.821530	25
FreshKills West	36-085-0111	40.580270	-74.198320	3
Pfizer Lab	36-005-0133	40.867900	-73.878090	31
Flax Pond	36-103-0044	40.961017	-73.139046	9
Rockland County	36-087-0005	41.182080	-74.028190	140
Millbrook	36-027-0007	41.785550	-73.741360	227

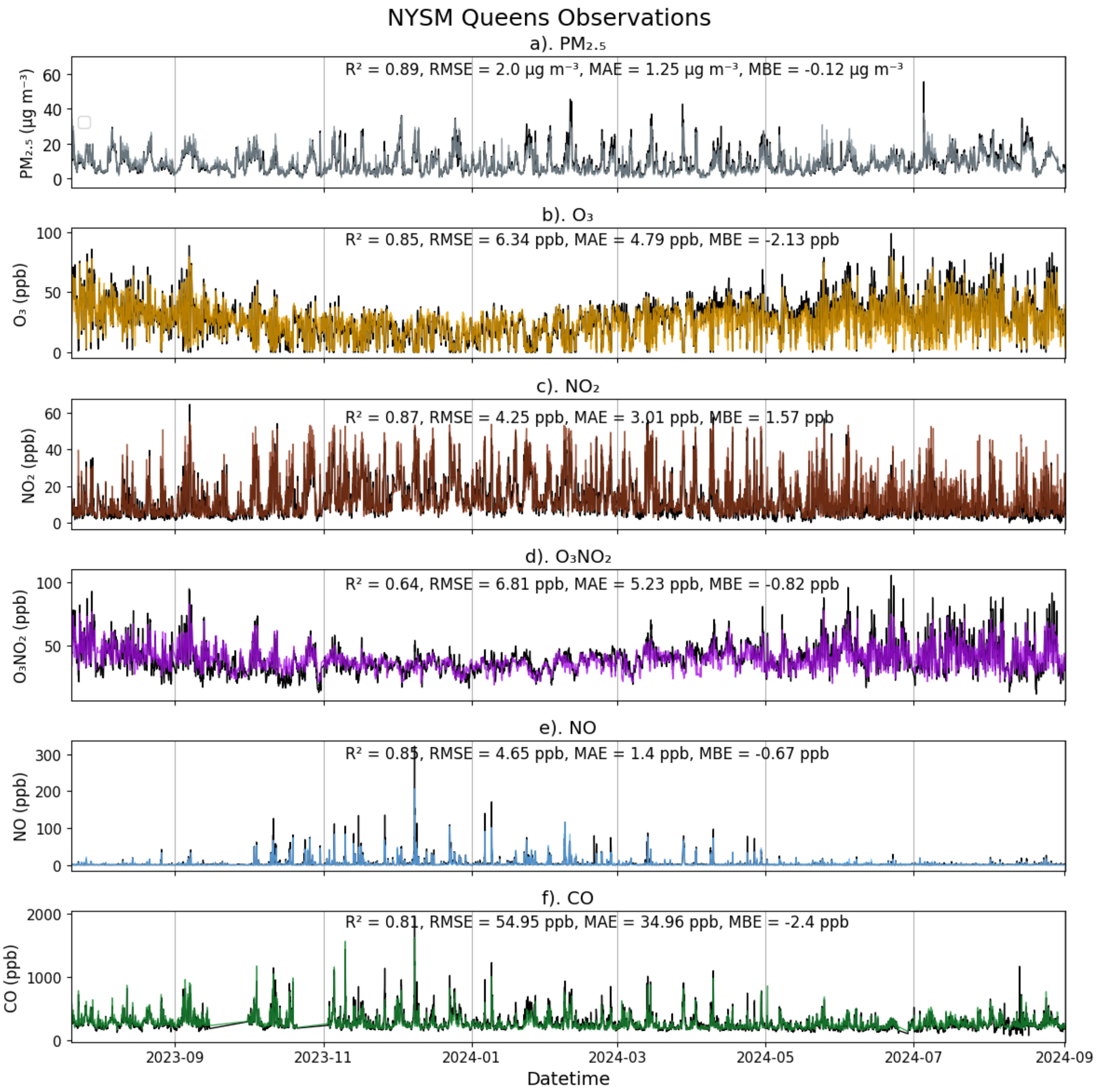

Figure S12. Hourly NCA-calibrated data for all six pollutants at the NYSM Queens site (various colors) plotted with simultaneous observations from the reference monitors (black) from the NYSDEC Queens College site (see Figure S2 for spatial information).

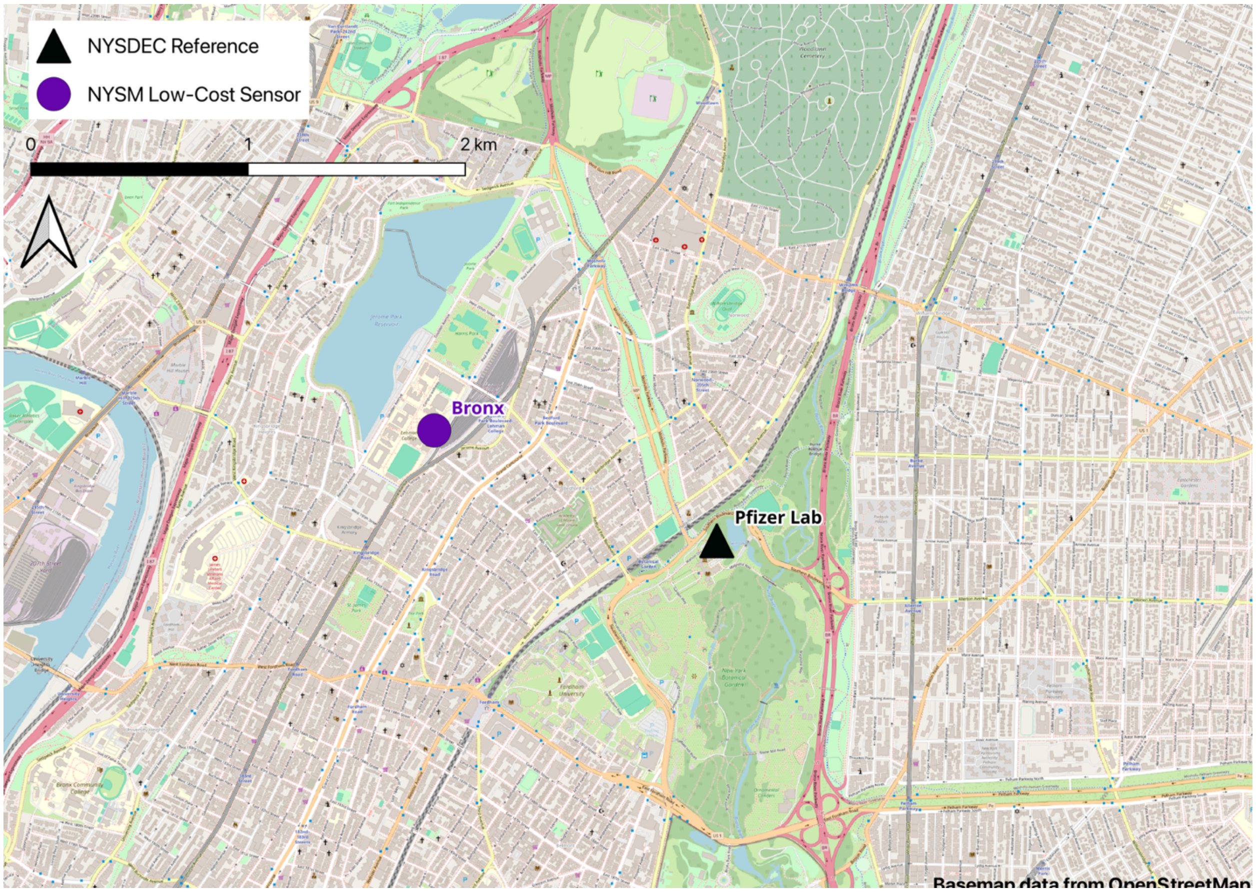

Figure S13. Map depicting the NYSDEC Pfizer Lab comparison site (black triangle) and the NYSM Bronx site (purple dot). The sites are approximately 1.4 km apart, however, the difference in surrounding land-use is remarkable. The NYSDEC Pfizer Lab site is located on a roof, surrounded by trees and a small pond whereas the NYSM Bronx site is located on the roof of the Lehman Center for Performing Arts on the CUNY Lehman College campus. The NYSM Bronx site is located in close proximity to a train station and the Jerome Park reservoir. The basemap was sourced from OpenStreetMap and the map was created in QGIS.

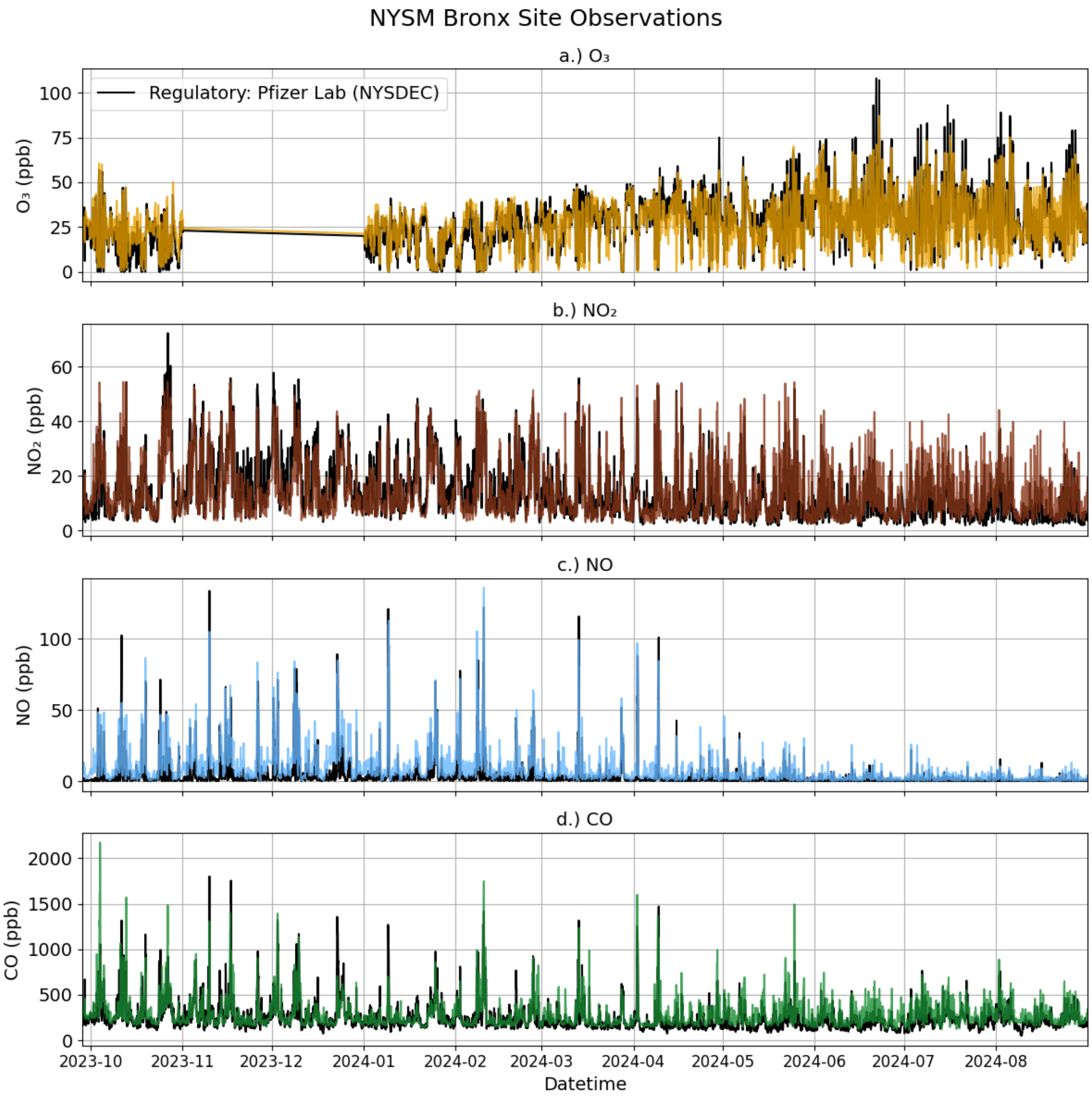

Figure S14. Hourly NCA-calibrated data for O_3 (a), NO_2 (b), NO (c), and CO (d) at the NYSM Bronx site (various colors) plotted with simultaneous observations from the reference monitors (black) from the NYSDEC Pfizer Lab site (see Figure S13 for spatial information). Reference data for O_3 in (a) from November to December 2023 were unavailable from the EPA AQS API.

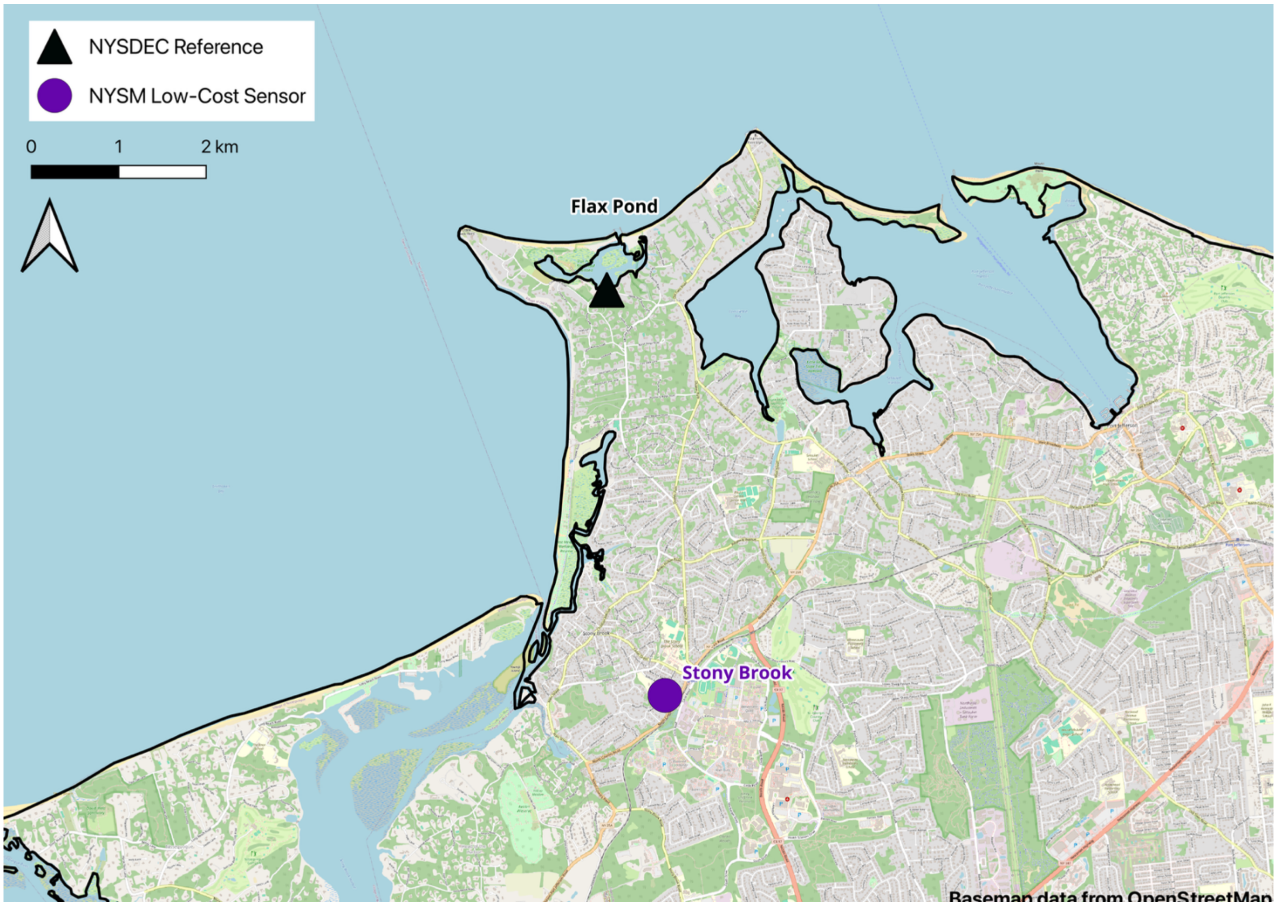

Figure S15. Map depicting the NYSDEC Flax Pond comparison site (black triangle) and the NYSM Stony Brook site (purple dot). The sites are approximately 4.7 km apart. The NYSDEC Flax Pond site is located on the north shore of Long Island and the south shore of Flax Pond, in a rural area. The NYSM Stony Brook site is located around 0.15 km from NYS Route 25A near a school and commercial area. The basemap was sourced from OpenStreetMap and the map was created in QGIS.

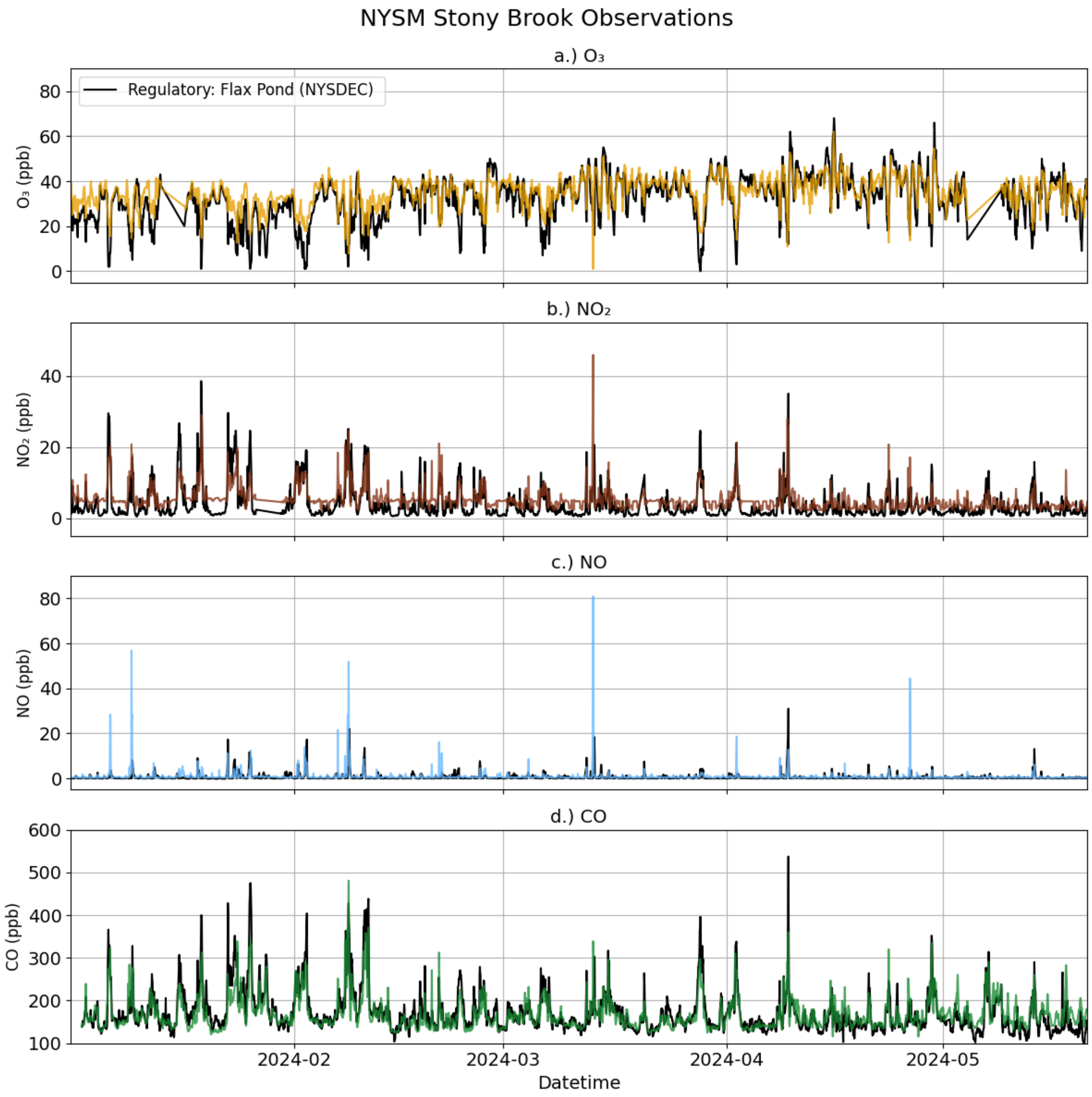

Figure S16. Hourly NCA-calibrated data for O_3 (a), NO_2 (b), NO (c), and CO (d) at the NYSM Stony Brook site (various colors) plotted with simultaneous observations from the reference monitors (black) from the NYSDEC Flax Pond site (see Figure S15 for spatial information).

Table S6. Calibration model performance on the network. The location of the NYSM Queens and NYSDEC Queens College sites are described in Section 2.2. The NYSM Stony Brook and NYSDEC Flax Pond sites are described in Section 3.3 and the figure captions of Figure S13 and Figure S15, respectively. The NYSM Suffern site is located on the Rockland Community College campus, 0.05km south of a soccer field. The site is located about 0.10 km north of residential housing. The NYSDEC Rockland County site is located in a rural field. The NYSM Dover Plains site is on a field, with NYS Route 343 located about 0.10 km east of the site. The NYSDEC Millbrook site is located on a field with two residential roads within 0.20 km of the site. Data from these sites were retrieved from the EPA AQS based on the site IDs provided in Table S5.

Site Pair	NYSDEC Regulatory Site	NYSM Site	Distance (km)	Pollutant	R^2	RMSE	MAE	MBE
1	Queens College	Queens (QUEE)	0.57 km	$PM_{2.5}$	0.89	2.0 µg m^{-3}	1.3 µg m^{-3}	-0.1 µg m^{-3}
				O_3	0.85	6.3 ppb	4.8 ppb	-2.1 ppb
				NO_2	0.87	4.3 ppb	3.0 ppb	1.6 ppb
				NO	0.85	4.7 ppb	1.4 ppb	-0.7 ppb
				CO	0.81	55.0 ppb	35.0 ppb	-2.4 ppb
2	FreshKills West	Fresh Kills (SIFKIL)	1.00 km	O_3	0.83	7.3 ppb	5.5 µg m^{-3}	1.7 µg m^{-3}
3	Pfizer Lab	Bronx (BRON)	1.40 km	O_3	0.74	7.7 ppb	5.9 ppb	-1.5 ppb
				NO_2	0.59	6.0 ppb	4.3 ppb	0.5 ppb
				NO	0.74	7.4 ppb	4.9 ppb	4.5 ppb
				CO	0.59	108.9 ppb	70.0 ppb	39.0 ppb
4	Flax Pond	Stony Brook (STON)	4.71 km	O_3	0.70	7.3 ppb	5.9 ppb	4.2 ppb
				NO_2	0.55	3.3 ppb	2.7 ppb	1.8 ppb
				NO	0.06	2.6 ppb	0.8 ppb	0.5 ppb
				CO	0.49	49.1 ppb	34.3 ppb	17.2 ppb
5	Rockland County	Suffern (SUFF)	7.74 km	O_3	0.61	8.9 ppb	6.8 ppb	1.9 ppb
6	Millbrook	Dover Plains (DOVE)	13.86 km	O_3	0.49	10.7 ppb	8.1 ppb	0.2 ppb
				NO_2	0.13	10.4 ppb	7.5 ppb	-5.8 ppb
				NO	0.21	13.7 ppb	5.1 ppb	-2.8 ppb

Section S7: Comparison with similar studies (Table S7-S11)

The following tables compare performance statistics of co-locations 1 and 2 (Table 2) to similar studies. If multiple models are evaluated in a study, the most similar model to the NCA NCM models was selected. The number of packages and/or co-locations is indicated under the 'N' column. The 'Reference Concentrations' column includes the average, median, and/or range of reference concentrations as reported. If a performance metric is not included in a study, it is indicated as 'NR' or 'not reported'. Results from Hojeily et al. 2025 are presented as the average ± the 95% confidence interval. Numeric superscripts indicate the reference number in the main text bibliography while alphabet superscripts refer to table footnotes. Performance for Hojeily et al. include only data validated at the NYSDEC Queens College site.

Table S7. $PM_{2.5}$ NCA performance comparison with similar studies.

Reference	Location	Sensor (Package)	Study Period	Temporal Resolution	Reference Concentrations (μg m⁻³)	Method	N	R^2	RMSE (μg m⁻³)	MAE (μg m⁻³)	MBE (μg m⁻³)
**Hojeily et al. (2025)**	**Queens, NY**	**Plantower PMS5003**	**8 April 2023 – 31 August 2024**	**1-hour**	**9.3 ± 0.3**	**Hybrid (network)**	**82**	**0.90 ± 0.01**	**1.8 ± 0.1**	**1.3 ± 0.1**	**-0.0 ± 0.2**
**Hojeily et al. (2025)**	**Queens, NY**	**Plantower PMS5003**	**5 June – 3 July 2023 (Wildfire)**	**1-hour**	**31.3 ± 1.8 (max. of 411)**	**Hybrid (network)**	**18**	**0.99 ± 0.00**	**18.2 ± 0.1**	**7.4 ± 0.0**	**-6.7 ± 0.0**
Levy Zamora et al. (2022)[5]	Balitmore, MD	Plantower PMSA003	1 February 2019 - 1 February 2020	**1-hour**	Median: 7.0 Average: 8.8	Spline MLR (individual)	1	0.72[a]	3.4	NR	NR
Barkjohn et al. (2021)[18, b]	United States	Plantower PMS5003 (PurpleAir)	September 2017 - January 2020	24-hour	Median range: 4-10	MLR (general)	53	0.83 Range: 0.81-0.85	Median: 2.5 Range: 1.8-5.5	Median: 1.8 Range: 1.4-3.2	Median: 0 Range: -0.9 – 1.3
Raheja et al. (2023)[33, c]	Accra, Ghana	Plantower PMS5003 (PurpleAir)	11 May -25 September, August 2021	1-hour	Range: 5.2 - 60	Gaussian Mixture Regression	2	0.86	2.6	1.9	NR
						RF	2	0.89	2.5	1.9	NR
						MLR	2	0.85	2.9	2.8	NR
						XGBoost	2	0.97	1.2	0.6	NR
deSouza et al. (2022)[41, d]	Denver, CO	Plantower PMS5003 (Love My Air)	1 January 2021 - 30 September 2021	1-hour	Median range: 10-11 at 5 co-located sites	MLR (Entire dataset correction)	5	0.93	3.4	NR	NR
						RF (Entire dataset correction)		0.98	1.7	NR	NR

[a] = the Pearson 'r' value of 0.85 is reported in Table 1 by Levy Zamora et al. (2019) which was converted to R^2
[b] = statistics retrieved from Barkjohn et al. (2021) Table S8 for the leave out by date validation for the '2+RH' correction model.
[c] = statistics retrieved from Raheja et al. (2023) Table 3 for their PurpleAir monitors.
[d] = statistics retrieved from their 'C1' models (deSouza et al. (2022), Table 2); the MLR model #2 with an RH predictor and the RF model #17 with predictors of temperature and RH. Five sites were co-located in their 'Love My Air' Network, with 24 additional field sites.

Table S8. O_3 NCA performance comparison with similar studies.

Reference	Location	Sensor (Package)	Study Period	Temporal Resolution	Reference Concentrations (ppb)	Method	N	R^2	RMSE (ppb)	MAE (ppb)	MBE (ppb)
**Hojeily et al. (2025)**	**Queens, NY**	**Alphasense OX-B431**	**8 April 2023 – 31 August 2024**	**1-hour**	**33.5 ± 1.2**	**Hybrid (network)**	**82**	**0.84 ± 0.02**	**6.4 ± 0.3**	**4.9 ± 0.3**	**-1.1 ± 0.5**
Levy Zamora et al. (2022)[5]	Essex, MD	MiCS-2614 (SEARCH)	1 February 2019 - 19 December 2019	1-hour	Median: 28.0 Average: 28.5	Spline MLR (individual)	1	0.79[a]	7.0	NR	NR
Malings et al. (2019)[10]	Pittsburgh, PA	Alphasense OX-B431 (RAMP)	Upwards of 18 months from 2016-2017	15-minute	Average range: 21-48	Hybrid (general)[b]	68	0.73 ± 0.12	NR	5.9 ± 1.5	0.9 ± 2.9
						Hybrid (individual)[c]	44	0.81 ± 0.09	NR	4.9 ± 1.3	-0.4 ± 1.5
Zimmerman et al. (2018)[7]	Pittsburgh, PA	Alphasense OX-B431 (RAMP)	3 August 2016 – 7 February 2017	15-minute	Average: 22	RF (individual)[d]	19	0.86 ± 0.02	NR	3.36 ± 0.41	-0.14 ± 0.46
Winter et al. (2025)[12,e]	Bay Area, CA	Alphasense OX-B431 (BEACO$_2$N)	2021 (365 days)	1-hour	Average: 26.8	MLR (individual, co-located)	1	0.95	2.48	1.91	-0.29
						MLR (individual, remotely calibrated)	1	0.88	3.70	2.94	0.02
Cross et al (2017)[40]	Boston, MA	Alphasense OX-B421 (ARISense)	7 July – 23 November 2016	5-min	Average: 23.2	High-dimensional model representation (HDMR)	2	0.39	9.71	7.34	0.78

[a] = the Pearson 'r' value of 0.89 is reported in Table 1 by Levy Zamora et al. (2019) which was converted to R^2.
[b] = statistics are taken from Malings et al. 2019 Table S4 for their 'HY' model, results are presented as the average ± the standard deviation.
[c] = statistics are taken from Malings et al. 2019 Table 5 for their 'HY' model, results are presented as the average ± the standard deviation.
[d] = Results presented as the average ± the standard deviation.
[e] = The total BEACO$_2$N network consisted of 4 co-located sites and over 50 field site deployments. Results are taken from their Table 3.

Table S9. NO_2 NCA performance comparison with similar studies.

Reference	Location	Sensor (Package)	Study Period	Temporal Resolution	Reference Concentrations (ppb)	Method	N	R^2	RMSE (ppb)	MAE (ppb)	MBE (ppb)
**Hojeily et al. (2025)**	**Queens, NY**	**Alphasense NO2-B43F**	**8 April 2023 – 31 August 2024**	**1-hour**	**11.1 ± 0.5**	**Hybrid (network)**	**82**	**0.82 ± 0.02**	**3.9 ± 0.2**	**2.8 ± 0.2**	**0.1 ± 0.3**
Levy Zamora et al. (2022)[5]	Essex, MD	Alphasense NO2-A43F (SEARCH)	1 February 2019 - 19 December 2019	1-hour	Median: 5.0 Average: 8.1	Spline MLR (individual)	1	0.77[a]	3.6	NR	NR
Malings et al. (2019)[10]	Pittsburgh, PA	Alphasense OX-B431 (RAMP)	Upwards of 18 months from 2016-2017	15-minute	Average range: 4-9	Hybrid (general)[b]	62	0.30 ± 0.17	NR	3.4 ± 0.5	0.2 ± 2.6
						Hybrid (individual)[c]	68	0.48 ± 0.10	NR	2.6 ± 0.4	0.5 ± 0.6
Zimmerman et al. (2018)[7]	Pittsburgh, PA	Alphasense OX-B431 (RAMP)	3 August 2016 – 7 February 2017	15-minute	Average: 12	RF (individual)[d]	16	0.67 ± 0.12	NR	3.48 ± 0.36	-0.4 ± 1.13
Winter et al. (2025)[12,e]	Bay Area, CA	Alphasense NO2-B43F (BEACO$_2$N)	2021 (365 days)	1-hour	Average: 6.4	MLR (individual, co-located)	1	0.82	2.26	1.72	0.31
						MLR (individual, remotely calibrated)	1	0.66	3.16	2.43	0.85
Cross et al. (2017)[40]	Boston, MA	Alphasense NO2-B43F (ARISense)	7 July – 23 November 2016	5-min	Average: 11.7	High-dimensional model representation (HDMR)	2	0.69	4.56	3.45	1.20

[a] = the Pearson 'r' value of 0. is reported in Table 1 by Levy Zamora et al. (2019) which was converted to R^2.
[b] = statistics are taken from Malings et al. 2019 Table S4 for their 'HY' model, results are presented as the average ± the standard deviation.
[c] = statistics are taken from Malings et al. 2019 Table 5 for their 'HY' model, results are presented as the average ± the standard deviation.
[d] = Results presented as the average ± the standard deviation.
[e] = The total BEACO$_2$N network consisted of 4 co-located sites and over 50 field site deployments. Results are taken from their Table 3.

Table S10. NO NCA performance comparison with similar studies.

Reference	Location	Sensor (Package)	Study Period	Temporal Resolution	Reference Concentrations (ppb)	Method	N	R^2	RMSE (ppb)	MAE (ppb)	MBE (ppb)
**Hojeily et al. (2025)**	**Queens, NY**	**Alphasense NO-B4**	**8 April 2023 – 31 August 2024**	**1-hour**	**2.4 ± 0.4**	**Hybrid (network)**	**82**	**0.81 ± 0.04**	**2.2 ± 0.2**	**1.2 ± 0.1**	**0.4 ± 0.2**
Levy Zamora et al. (2022)[5]	Essex, MD	Alphasense NO-A4 (SEARCH)	1 February 2019 - 19 December 2020	1-hour	Median: 0.6 Average: 3.3	Spline MLR (individual)	1	0.94[a]	3.2	NR	NR
Malings et al. (2019)[10]	Pittsburgh, PA	Alphasense OX-B431 (RAMP)	Upwards of 18 months from 2016-2017	15-minute	Average range: 1-4	Hybrid (general)[b]	20	0.17 ± 0.08	NR	13.2 ± 22.7	9.9 ± 20.1
						Hybrid (individual)[3]	19	0.32 ± 0.14	NR	2.3 ± 0.8	0.8 ± 0.7
Winter et al. (2025)[12,e]	Bay Area, CA	Alphasense NO-B4 ($BEACO_2N$)	2021 (365 days)	1-hour	Average: 2.4	MLR (individual, co-located)	1	0.82	1.46	1.00	0.30
						MLR (individual, remotely calibrated)	1	0.79	1.58	1.08	0.39
Cross et al. (2017)[40]	Boston, MA	Alphasense NO-B4 (ARISense)	July - November 2016 (88 days)	5-min	Average: 6.1	High-dimensional model representation (HDMR)	2	0.84	4.52	2.83	0.97

[a] = the Pearson 'r' value of 0.97 is reported in Table 1 of Levy Zamora et al. (2019) which was converted to R^2.
[b] = statistics are taken from Malings et al. 2019 Table S4 for their 'HY' model, results are presented as the average ± the standard deviation.
[c] = statistics are taken from Malings et al. 2019 Table 5 for their 'HY' model, results are presented as the average ± the standard deviation.
[e] = The total $BEACO_2N$ network consisted of 4 co-located sites and over 50 field site deployments. Results are taken from their Table 3.

Table S11. CO NCA performance comparison with similar studies.

Reference	Location	Sensor (Package)	Study Period	Temporal Resolution	Reference Concentrations (ppb)	Method	N	R^2	RMSE (ppb)	MAE (ppb)	MBE (ppb)
**Hojeily et al. 2025**	**Queens, NY**	**Alphasense CO-B4**	**8 April 2023 – 31 August 2024**	**1-hour**	**259.9 ± 6.7**	**Linear Quadratic (network)**	**82**	**0.87 ± 0.03**	**46.4 ± 2.4**	**35.2 ± 1.0**	**9.4 ± 5.7**
Levy Zamora et al. (2022)[5]	Essex, MD	Alphasense CO-A4 (SEARCH)	1 February 2019 – 1 February 2020	1-hour	Median: 203<br>Average: 282	Spline MLR (individual)	1	0.94[a]	58.9	NR	NR
Malings et al. (2019)[10]	Pittsburgh, PA	Alphasense OX-B431 (RAMP)	Upwards of 18 months from 2016-2017	15-minute	Average range:145-451	Linear Quadratic Regression (general)[b]	48	0.85 ± 0.09	NR	56 ± 8	6 ± 93
						Linear Quadratic Regression (individual)[c]	68	0.83 ± 0.10	NR	48 ± 11	-5 ± 14
Zimmerman et al. (2018)[7]	Pittsburgh, PA	Alphasense OX-B431 (RAMP)	3 August 2016 – 7 February 2017	15-minute	Average: 270	RF (individual)[4]	19	0.91 ± 0.05	NR	38.0 ± 6.5	0.1 ± 0.20
Winter et al. (2025)[12,e]	Bay Area, CA	Alphasense CO-B4 (BEACO$_2$N)	2023 (365 days)	1-hour	Average: 158	MLR (individual, co-located)	1	0.90	33.4	20.2	1.50
Cross et al. (2017)[40]	Boston, MA	Alphasense CO-B4 (ARISense)	7 July – 23 November 2016	5-min	Average: 231	High-dimensional model representation (HDMR)	2	0.88	39.2	24.8	-10.4

[a] = the Pearson 'r' value of 0.97 is reported in Table 1 by Levy Zamora et al. (2019) which was converted to R^2
[b] = statistics are taken from Malings et al. 2019 Table S4 for their 'HY' model, results are presented as the average ± the standard deviation.
[c] = statistics are taken from Malings et al. 2019 Table 5 for their 'HY' model, results are presented as the average ± the standard deviation.
[d] = Results presented as the average ± the standard deviation.
[e] = The total BEACO$_2$N network consisted of 4 co-located sites and over 50 field site deployments. Results are taken from their Table 3.
