# Supplementary material for: Correction to “A Network Calibration Approach Improves the Accuracy and Long-Term Stability of a Low-Cost Air Quality Mesonet in New York City”
Source: ACS EST Air. 2026 Sep 28;3(10):2909–10. doi: 10.1021/acsestair.6c00297 (PMC13660386; doi:10.1021/acsestair.6c00297)
Supplement: Supplementary file 1 [file ea6c00297_si_001.pdf]

# Supplemental Information

**Correction to "A Network Calibration Approach Improves the Accuracy and Long-Term Stability of a Low-Cost Air Quality Mesonet in New York City"**

Ellie H. Hojeily, Jason M. Covert, Margaret J. Schwab, Clover Moore, Cheng-Hsuan Lu, Md. Aynul Bari, Scott D. Miller

Original citation:

Hojeily, E. H.; Covert, J. M.; Schwab, M. J.; Moore, C.; Lu, C.-H.; Bari, Md. A.; Miller, S. D. A Network Calibration Approach Improves the Accuracy and Long-Term Stability of a Low-Cost Air Quality Mesonet in New York City. *ACS EST Air* **2026**, *3* (1), 58–72. https://doi.org/10.1021/acsestair.5c00205.

<u>Section S7: Comparison with similar studies (Table S7-S11)</u>

**Table S7**. $PM_{2.5}$ NCA performance comparison with similar studies.

| Reference | Location | Sensor (Package) | Study Period | Temporal Resolution | Reference Concentrations ($\mu g\ m^{-3}$) | Method | N | $r^2$ | $R^2$ | RMSE ($\mu g\ m^{-3}$) | MAE ($\mu g\ m^{-3}$) | MBE ($\mu g\ m^{-3}$) |
|---|---|---|---|---|---|---|---|---|---|---|---|---|
| **Hojeily et al. (2025)** | **Queens, NY** | **Plantower PMS5003** | **8 April 2023 – 31 August 2024** | **1-hour** | **9.3 ± 0.3** | **Hybrid (network)** | **82** | **0.90 ± 0.01** | **0.88 ± 0.02** | **1.8 ± 0.1** | **1.3 ± 0.1** | **-0.0 ± 0.2** |
| **Hojeily et al. (2025)** | **Queens, NY** | **Plantower PMS5003** | **5 June – 3 July 2023 (Wildfire)** | **1-hour** | **31.3 ± 1.8 (max. of 411)** | **Hybrid (network)** | **18** | **0.99 ± 0.00** | **0.86 ± 0.00** | **18.2 ± 0.1** | **7.4 ± 0.0** | **-6.7 ± 0.0** |
| Levy Zamora et al. (2022)[5] | Balitmore, MD | Plantower PMSA003 | 1 February 2019 - 1 February 2020 | **1-hour** | Median: 7.0 Average: 8.8 | Spline MLR (individual) | 1 | 0.72[1] | NR | 3.4 | NR | NR |
| Barkjohn et al. (2021)[18, b] | United States | Plantower PMS5003 (PurpleAir) | September 2017 - January 2020 | 24-hour | Median range: 4-10 | MLR (general) | 53 | NR | 0.83 Range: 0.81-0.85 | Median: 2.5 Range: 1.8-5.5 | Median: 1.8 Range: 1.4-3.2 | Median: 0 Range: -0.9 – 1.3 |
| Raheja et al. (2023)[33, c] | Accra, Ghana | Plantower PMS5003 (PurpleAir) | 11 May -25 September, August 2021 | 1-hour | Range: 5.2 - 60 | Gaussian Mixture Regression | 2 | NR | 0.86 | 2.6 | 1.9 | NR |
| | | | | | | RF | 2 | NR | 0.89 | 2.5 | 1.9 | NR |
| | | | | | | MLR | 2 | NR | 0.85 | 2.9 | 2.8 | NR |
| | | | | | | XGBoost | 2 | NR | 0.97 | 1.2 | 0.6 | NR |
| deSouza et al. (2022)[41, d] | Denver, CO | Plantower PMS5003 (Love My Air) | 1 January 2021 - 30 September 2021 | 1-hour | Median range: 10-11 at 5 co-located sites | MLR (Entire dataset correction) | 5 | NR | 0.93 | 3.4 | NR | NR |
| | | | | | | RF (Entire dataset correction) | | NR | 0.98 | 1.7 | NR | NR |

[a] = the Pearson ‘r’ value of 0.85 is reported in Table 1 by Levy Zamora et al. (2019) which was converted to $r^2$
[b] = statistics retrieved from Barkjohn et al. (2021) Table S8 for the leave out by date validation for the ‘2+RH’ correction model.
[c] = statistics retrieved from Raheja et al. (2023) Table 3 for their PurpleAir monitors.
[d] = statistics retrieved from their ‘C1’ models (deSouza et al. (2022), Table 2); the MLR model #2 with an RH predictor and the RF model #17 with predictors of temperature and RH. Five sites were co-located in their ‘Love My Air’ Network, with 24 additional field sites. Pearson correlation R reported

**Table S8**. $O_3$ NCA performance comparison with similar studies.

| Reference | Location | Sensor (Package) | Study Period | Temporal Resolution | Reference Concentrations (ppb) | Method | N | $r^2$ | $R^2$ | RMSE (ppb) | MAE (ppb) | MBE (ppb) |
|---|---|---|---|---|---|---|---|---|---|---|---|---|
| **Hojeily et al. (2025)** | **Queens, NY** | **Alphasense OX-B431** | **8 April 2023 – 31 August 2024** | **1-hour** | **33.5 ± 1.2** | **Hybrid (network)** | **82** | **0.84 ± 0.02** | **0.79 ± 0.02** | **6.4 ± 0.3** | **4.9 ± 0.3** | **-1.1 ± 0.5** |
| Levy Zamora et al. (2022)[5] | Essex, MD | MiCS-2614 (SEARCH) | 1 February 2019 - 19 December 2019 | 1-hour | Median: 28.0 Average: 28.5 | Spline MLR (individual) | 1 | 0.79[a] | NR | 7.0 | NR | NR |
| Malings et al. (2019)[10] | Pittsburgh, PA | Alphasense OX-B431 (RAMP) | Upwards of 18 months from 2016-2017 | 15-minute | Average range: 21-48 | Hybrid (general)[b] | 68 | 0.73 ± 0.12 | NR | NR | 5.9 ± 1.5 | 0.9 ± 2.9 |
| | | | | | | Hybrid (individual)[c] | 44 | 0.81 ± 0.09 | NR | NR | 4.9 ± 1.3 | -0.4 ± 1.5 |
| Zimmerman et al. (2018)[7] | Pittsburgh, PA | Alphasense OX-B431 (RAMP) | 3 August 2016 – 7 February 2017 | 15-minute | Average: 22 | RF (individual)[d] | 19 | NR | 0.86 ± 0.02 | NR | 3.36 ± 0.41 | -0.14 ± 0.46 |
| Winter et al. (2025)[12,e] | Bay Area, CA | Alphasense OX-B431 ($BEACO_2N$) | 2021 (365 days) | 1-hour | Average: 26.8 | MLR (individual, co-located) | 1 | NR | 0.95 | 2.48 | 1.91 | -0.29 |
| | | | | | | MLR (individual, remotely calibrated) | 1 | NR | 0.88 | 3.70 | 2.94 | 0.02 |
| Cross et al (2017)[40] | Boston, MA | Alphasense OX-B421 (ARISense) | 7 July – 23 November 2016 | 5-min | Average: 23.2 | High-dimensional model representation (HDMR) | 2 | NR | 0.39 | 9.71 | 7.34 | 0.78 |

[a] = the Pearson 'r' value of 0.89 is reported in Table 1 by Levy Zamora et al. (2019) which was converted to $r^2$.
[b] = statistics are taken from Malings et al. 2019 Table S4 for their 'HY' model, results are presented as the average ± the standard deviation. For $R^2$, their $r^2$ is reported
[c] = statistics are taken from Malings et al. 2019 Table 5 for their 'HY' model, results are presented as the average ± the standard deviation. For $R^2$, their $r^2$ is reported
[d] = Results presented as the average ± the standard deviation.
[e] = The total $BEACO_2N$ network consisted of 4 co-located sites and over 50 field site deployments. Results are taken from their Table 3.

**Table S9**. $NO_2$ NCA performance comparison with similar studies.

| Reference | Location | Sensor | Study Period | Temporal Resolution | Reference Concentrations (ppb) | Method | N | $r^2$ | $R^2$ | RMSE (ppb) | MAE (ppb) | MBE (ppb) |
|---|---|---|---|---|---|---|---|---|---|---|---|---|
| **Hojeily et al. (2025)** | **Queens, NY** | **Alphasense NO2-B43F** | **8 April 2023 – 31 August 2024** | **1-hour** | **11.1 ± 0.5** | **Hybrid (network)** | **82** | **0.82 ± 0.02** | **0.77 ± 0.04** | **3.9 ± 0.2** | **2.8 ± 0.2** | **0.1 ± 0.3** |
| Levy Zamora et al. (2022)[5] | Essex, MD | Alphasense NO2-A43F (SEARCH) | 1 February 2019 - 19 December 2019 | 1-hour | Median: 5.0 Average: 8.1 | Spline MLR (individual) | 1 | 0.77[a] | NR | 3.6 | NR | NR |
| Malings et al. (2019)[10] | Pittsburgh, PA | Alphasense OX-B431 (RAMP) | Upwards of 18 months from 2016-2017 | 15-minute | Average range: 4-9 | Hybrid (general)[b] | 62 | 0.30 ± 0.17 | NR | NR | 3.4 ± 0.5 | 0.2 ± 2.6 |
| | | | | | | Hybrid (individual)[c] | 68 | 0.48 ± 0.10 | NR | NR | 2.6 ± 0.4 | 0.5 ± 0.6 |
| Zimmerman et al. (2018)[7] | Pittsburgh, PA | Alphasense OX-B431 (RAMP) | 3 August 2016 – 7 February 2017 | 15-minute | Average: 12 | RF (individual)[d] | 16 | NR | 0.67 ± 0.12 | NR | 3.48 ± 0.36 | -0.4 ± 1.13 |
| Winter et al. (2025)[12,e] | Bay Area, CA | Alphasense NO2-B43F ($BEACO_2N$) | 2021 (365 days) | 1-hour | Average: 6.4 | MLR (individual, co-located) | 1 | NR | 0.82 | 2.26 | 1.72 | 0.31 |
| | | | | | | MLR (individual, remotely calibrated) | 1 | NR | 0.66 | 3.16 | 2.43 | 0.85 |
| Cross et al. (2017)[40] | Boston, MA | Alphasense NO2-B43F (ARISense) | 7 July – 23 November 2016 | 5-min | Average: 11.7 | High-dimensional model representation (HDMR) | 2 | NR | 0.69 | 4.56 | 3.45 | 1.20 |

[a] = the Pearson ‘r’ value of 0.89 is reported in Table 1 by Levy Zamora et al. (2019) which was converted to $r^2$.
[b] = statistics are taken from Malings et al. 2019 Table S4 for their ‘HY’ model, results are presented as the average ± the standard deviation. For $R^2$, their $r^2$ is reported
[c] = statistics are taken from Malings et al. 2019 Table 5 for their ‘HY’ model, results are presented as the average ± the standard deviation. For $R^2$, their $r^2$ is reported
[d] = Results presented as the average ± the standard deviation.
[e] = The total $BEACO_2N$ network consisted of 4 co-located sites and over 50 field site deployments. Results are taken from their Table 3.

**Table S10**. NO NCA performance comparison with similar studies.

| Reference | Location | Sensor (Package) | Study Period | Temporal Resolution | Reference Concentrations (ppb) | Method | N | $r^2$ | $R^2$ | RMSE (ppb) | MAE (ppb) | MBE (ppb) |
|---|---|---|---|---|---|---|---|---|---|---|---|---|
| **Hojeily et al. (2025)** | **Queens, NY** | **Alphasense NO-B4** | **8 April 2023 – 31 August 2024** | **1-hour** | **2.4 ± 0.4** | **Hybrid (network)** | **82** | **0.81 ± 0.04** | **0.74 ± 0.04** | **2.2 ± 0.2** | **1.2 ± 0.1** | **0.4 ± 0.2** |
| Levy Zamora et al. (2022)[5] | Essex, MD | Alphasense NO-A4 (SEARCH) | 1 February 2019 - 19 December 2020 | 1-hour | Median: 0.6<br>Average: 3.3 | Spline MLR (individual) | 1 | 0.94[a] | NR | 3.2 | NR | NR |
| Malings et al. (2019)[10] | Pittsburgh, PA | Alphasense OX-B431 (RAMP) | Upwards of 18 months from 2016-2017 | 15-minute | Average range: 1-4 | Hybrid (general)[b] | 20 | 0.17 ± 0.08 | NR | NR | 13.2 ± 22.7 | 9.9 ± 20.1 |
| | | | | | | Hybrid (individual)[3] | 19 | 0.32 ± 0.14 | NR | NR | 2.3 ± 0.8 | 0.8 ± 0.7 |
| Winter et al. (2025)[12,e] | Bay Area, CA | Alphasense NO-B4 ($BEACO_2N$) | 2021 (365 days) | 1-hour | Average: 2.4 | MLR (individual, co-located) | 1 | NR | 0.82 | 1.46 | 1.00 | 0.30 |
| | | | | | | MLR (individual, remotely calibrated) | 1 | NR | 0.79 | 1.58 | 1.08 | 0.39 |
| Cross et al. (2017)[40] | Boston, MA | Alphasense NO-B4 (ARISense) | July - November 2016 (88 days) | 5-min | Average: 6.1 | High-dimensional model representation (HDMR) | 2 | NR | 0.84 | 4.52 | 2.83 | 0.97 |

[a] = the Pearson 'r' value of 0.97 is reported in Table 1 of Levy Zamora et al. (2019) which was converted to $r^2$.
[b] = statistics are taken from Malings et al. 2019 Table S4 for their 'HY' model, results are presented as the average ± the standard deviation. . For $R^2$, their $r^2$ is reported
[c] = statistics are taken from Malings et al. 2019 Table 5 for their 'HY' model, results are presented as the average ± the standard deviation. . For $R^2$, their $r^2$ is reported
[e] = The total $BEACO_2N$ network consisted of 4 co-located sites and over 50 field site deployments. Results are taken from their Table 3.

**Table S11**. CO NCA performance comparison with similar studies.

| Reference | Location | Sensor (Package) | Study Period | Temporal Resolution | Reference Concentrations (ppb) | Method | N | $r^2$ | $R^2$ | RMSE (ppb) | MAE (ppb) | MBE (ppb) |
|---|---|---|---|---|---|---|---|---|---|---|---|---|
| **Hojeily et al. 2025** | **Queens, NY** | **Alphasense CO-B4** | **8 April 2023 – 31 August 2024** | **1-hour** | **259.9 ± 6.7** | **Linear Quadratic (network)** | **82** | **0.87 ± 0.03** | **0.81 ± 0.03** | **46.4 ± 2.4** | **35.2 ± 1.0** | **9.4 ± 5.7** |
| Levy Zamora et al. (2022)[5] | Essex, MD | Alphasense CO-A4 (SEARCH) | 1 February 2019 – 1 February 2020 | 1-hour | Median: 203 Average: 282 | Spline MLR (individual) | 1 | 0.94[a] | NR | 58.9 | NR | NR |
| Malings et al. (2019)[10] | Pittsburgh, PA | Alphasense OX-B431 (RAMP) | Upwards of 18 months from 2016-2017 | 15-minute | Average range:145-451 | Linear Quadratic Regression (general)[b] | 48 | 0.85 ± 0.09 | NR | NR | 56 ± 8 | 6 ± 93 |
| | | | | | | Linear Quadratic Regression (individual)[c] | 68 | 0.83 ± 0.10 | NR | NR | 48 ± 11 | -5 ± 14 |
| Zimmerman et al. (2018)[7] | Pittsburgh, PA | Alphasense OX-B431 (RAMP) | 3 August 2016 – 7 February 2017 | 15-minute | Average: 270 | RF (individual)[4] | 19 | NR | 0.91 ± 0.05 | NR | 38.0 ± 6.5 | 0.1 ± 0.20 |
| Winter et al. (2025)[12,e] | Bay Area, CA | Alphasense CO-B4 (BEA$CO_2$N) | 2023 (365 days) | 1-hour | Average: 158 | MLR (individual, co-located) | 1 | NR | 0.90 | 33.4 | 20.2 | 1.50 |
| Cross et al. (2017)[40] | Boston, MA | Alphasense CO-B4 (ARISense) | 7 July – 23 November 2016 | 5-min | Average: 231 | High-dimensional model representation (HDMR) | 2 | NR | 0.88 | 39.2 | 24.8 | -10.4 |

[a] = the Pearson 'r' value of 0.97 is reported in Table 1 by Levy Zamora et al. (2019) which was converted to $r^2$
[b] = statistics are taken from Malings et al. 2019 Table S4 for their 'HY' model, results are presented as the average ± the standard deviation. . For $R^2$, their $r^2$ is reported
[c] = statistics are taken from Malings et al. 2019 Table 5 for their 'HY' model, results are presented as the average ± the standard deviation. . For $R^2$, their $r^2$ is reported
[d] = Results presented as the average ± the standard deviation.
[e] = The total BEA$CO_2$N network consisted of 4 co-located sites and over 50 field site deployments. Results are taken from their Table 3.
